# Characterization of Three Novel 4-Methylaminorex Derivatives Applied as Designer Drugs

**DOI:** 10.3390/molecules27185770

**Published:** 2022-09-06

**Authors:** Elisabeth Seibert, Olaf Kunert, Eva-Maria Pferschy-Wenzig, Martin G. Schmid

**Affiliations:** 1 Department of Pharmaceutical Chemistry, Institute of Pharmaceutical Sciences, University of Graz, Schubertstraße 1, 8010 Graz, Austria; 2 Department of Pharmacognosy, Institute of Pharmaceutical Sciences, University of Graz, Beethovenstraße 8, 8010 Graz, Austria

**Keywords:** halogenated 4-methylaminorex derivatives, chiral separation, NMR, LC-HRMS, HPLC-UV, 4′-fluoro-4-methylaminorex, 4′-chloro-4-methylaminorex, 4′-bromo-4-methylaminorex

## Abstract

The ongoing development of more and more new psychoactive substances continues to be a huge problem in 2022 affecting the European and international drug market. Through slight alterations in the structure of illicit drugs, a way to circumvent the law is created, as the created derivatives serve as legal alternatives with similar effects. A common way of structure modification is the induction of a halogen residue. Recently, halogenated derivatives of the well-known designer drug 4-methylaminorex appeared on the market and are available in various online shops. In this study, three novel halogenated 4-methylaminorex derivatives, namely 4′-fluoro-4-methylaminorex, 4′-chloro-4-methylaminorex, and 4′-bromo-4-methylaminorex, were purchased online and characterized using nuclear magnetic resonance (NMR) spectroscopy, liquid chromatography-high-resolution mass spectrometry (LC-HRMS), and chiral high-performance liquid chromatography with ultraviolet detection (HPLC-UV). These derivatives possess two stereogenic centers, and analyses revealed that all of them were present as a racemic mixture of the *trans* diastereomeric form.

## 1. Introduction

For more than one decade, there has been a strong trend to replace classical illicit drugs with so-called new psychoactive substances (NPS) to circumvent legislation and to replace illicit substances with legal alternatives with similar psychotropic effects. These compounds are usually produced in clandestine labs, and were introduced in different generations. For example, cathinones representing a substance class similar to amphetamines were introduced in the late 2000s [1]. At times of low quality and high prices of cocaine in Europe, the most popular cathinone derivative called mephedrone (4-methyl-methcathinone) launched as an affordable alternative and gained unpredictable popularity [2]. To date, about 150 cathinone derivatives with slightly altered side chains have been created for the NPS market. Besides cathinones, further novel amphetamine derivatives have been synthesized and traded mainly via the internet to enlarge the portfolio of stimulants. Similarly, novel ketamine derivatives, tryptamines, and modified lysergic acid diethylamide (LSD) derivatives entered the global market between 2006 and 2022 [3]. In recent years, methylphenidate, a medicinal drug used to treat attention deficit hyperactivity disorder, has been replaced by changing the length of the molecule’s side chain, resulting in compounds like ethylphenidate and propylphenidate [4].

Another approach to altering molecule structure is introducing halogen moieties [5,6,7]. Due to the prohibition of the parent compounds amphetamine (“Speed”) and methamphetamine (“Crystal Meth”) half a century ago, the structures of these molecules have been altered by the introduction of fluorine or chlorine atoms, first in para, and later in meta and ortho position.

Recently, aminorex, an anorectic stimulant drug, underwent slight changes in its chemical structure. Developed in 1962, it belongs to the substance class of 2-amino-5-aryloxazolines and possesses a high potential for abuse [8]. It can be regarded as a metabolite of levamisole, a well-known adulterant added to cocaine of street quality [9]. Similar to amphetamine or cathinone, a methyl derivative called 4-methylaminorex (MAR) was synthesized as early as 1960 in the same scientific lab [10]. Since these parent compounds are scheduled in most countries, recently, three derivatives, namely 4′-fluoro-4-methylaminorex (4F-MAR), 4′-chloro-4-methylaminorex (4C-MAR), and 4′-bromo-4-methylaminorex (4B-MAR) have been developed as substitution products. These three novel NPS are characterized in this contribution since there is little knowledge about them. 4′-Fluoro-4-methylaminorex first came up in Slovenia in 2018 [11]. One year later, 2C-B-aminorex, structurally a hybrid of 4-bromo-2,5-dimethoxyphenethylamine (2C-B) and aminorex, was first reported to the European Monitoring Centre for Drugs and Drug Addiction (EMCDDA) from Sweden [12]. Also, further derivatives have been described in literature before [13,14]. Meanwhile, the 4′-chloro-derivative and its 4′-bromo analog are also commercially available on the internet. However, to the best of our knowledge, there is no chemical characterization of these two compounds in the literature.

At the end of 2021, EMCDDA was monitoring around 880 NPS; 52 of them were first reported in Europe in 2021, among them 224 synthetic cannabinoids, 162 cathinones, and 106 phenethylamines [15]. Since many of them possess a chiral center, it is not clear whether their psychotropic effect is related to just one enantiomer or whether the activity differs between the enantiomers. Almost nothing is known about potential toxicity because NPS are designed on the drawing board to replace their prohibited parent compounds and therefore lack clinical investigation. There are few reports about the enantioselective effects of NPS [1]. Many analytical approaches have been made to resolve chiral NPS of various substance classes into their enantiomers, such as chiral HPLC, gas chromatography, capillary electrophoresis, and capillary electrochromatography [16]. Their outcome revealed that most chiral NPS are traded as racemic mixtures.

**Figure 1:**
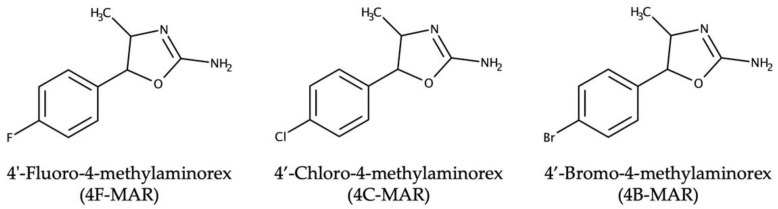
Chemical structures of the three novel 4-methylaminorex derivatives investigated.

The goal of the present contribution is to characterize the aforementioned three recent halogenated derivatives of 4-methylaminorex, as indicated in Figure 1. They were purchased from an internet provider in January 2022. To our best knowledge, these compounds are not yet available from serious chemical providers.

## 2. Results

Three novel halogenated 4-methylaminorex derivatives recently offered on the internet, obviously to substitute the illicit parent compounds aminorex and 4-methyl-aminorex, have been characterized by NMR spectroscopy, LC-HRMS analysis, and chiral HPLC-UV-analysis.

### 2.1. NMR Spectroscopy Measurements

The assignments of proton and carbon resonances of the three 4-methylaminorex derivatives were obtained by analyzing correlations in the COSY and HMBC spectra. The methyl protons were correlated in HMBC with C-4 and C-5, in the COSY spectrum with H-4. The methine protons H-4 and H-5 were correlated with C-2 in HMBC. Proton resonances of H-3′/5′ were then correlated in HMBC with C-5. Resonance assignments are given in Table 1 and Table 2. The assignments of proton and carbon resonances of the three analytes are given in Table 1 and Table 2, and spectra are displayed in Supplementary Materials (Figures S1–S21). The comparison of the proton and carbon shift values for positions C-2, C-4, and C-5 obtained in CDCl_3_ (Table 1) with literature data from both diastereomeric pairs of 4-methylaminorex [17] revealed that the compounds under investigation belonged to the *trans*-series of 4-methylaminorex derivatives, i.e., the methyl group and the phenyl moiety were on opposite faces of the heterocycle. Hence, the compounds have either 4*S*,5*S*-configuration or 4*R*,5*R*-configuration. Furthermore, purity of the compounds was determined by integrating methyl NMR resonances and was found to be 85% for 4F-MAR, 98% for 4C-MAR and 90% for 4B-MAR, respectively.

### 2.2. LC-HRMS Measurements

LC-HRMS analysis allowed the detection of the three compounds as protonated species in the HESI positive ion mode. The molecular formulas deduced from their *m*/*z* values (Table 3) were clearly in accordance with the structures suggested by NMR spectroscopy, and their isotopic patterns indicated the presence of bromine in 4B-MAR, chlorine in 4C-MAR and fluorine in 4F-MAR (for spectra, see Supplementary Materials, Figures S23–S25). In the MS/MS spectra of all three compounds (Supplementary Materials, Figures S26–S28), the main MS/MS fragment generated both with high and low collision energy indicated the loss of a CONH moiety, which is due to the cleavage of the amino-oxazoline ring present in all three substances (*m*/*z* 212.0068 for 4B-MAR, *m*/*z* 168.0573 for 4C-MAR and *m*/*z* 152.0870 for 4F-MAR). This fragment was also visible in the full MS spectra of the compounds (Figures S23–S25), indicating the occurrence of partial in-source fragmentation under the applied MS conditions. Further fragments that occurred at enhanced relative abundances when high collision energy was applied were also quite similar for all three compounds. In the case of 4B-MAR, they were due to an additional loss of Br (*m*/*z* 133.0887) and due to an additional loss of both NH_3_ and Br (*m*/*z* 116.0623). In the case of 4C-MAR, the additional loss of NH_3_ led to *m*/*z* 151.0309, the additional loss of Cl led to fragment *m*/*z* 133.0888, while the additional loss of both Cl and NH_3_ led to fragment *m*/*z* 116.0626 and the loss NH_3_ and HCl provided fragment *m*/*z* 115.0545. For 4F-MAR, the fragment at *m*/*z* 135.0606 was due to additional NH_3_ loss, and fragment *m*/*z* 115.0546 indicated the loss of NH_3_ and HF.

### 2.3. Experiments by Chiral HPLC-UV

**Figure 2:**
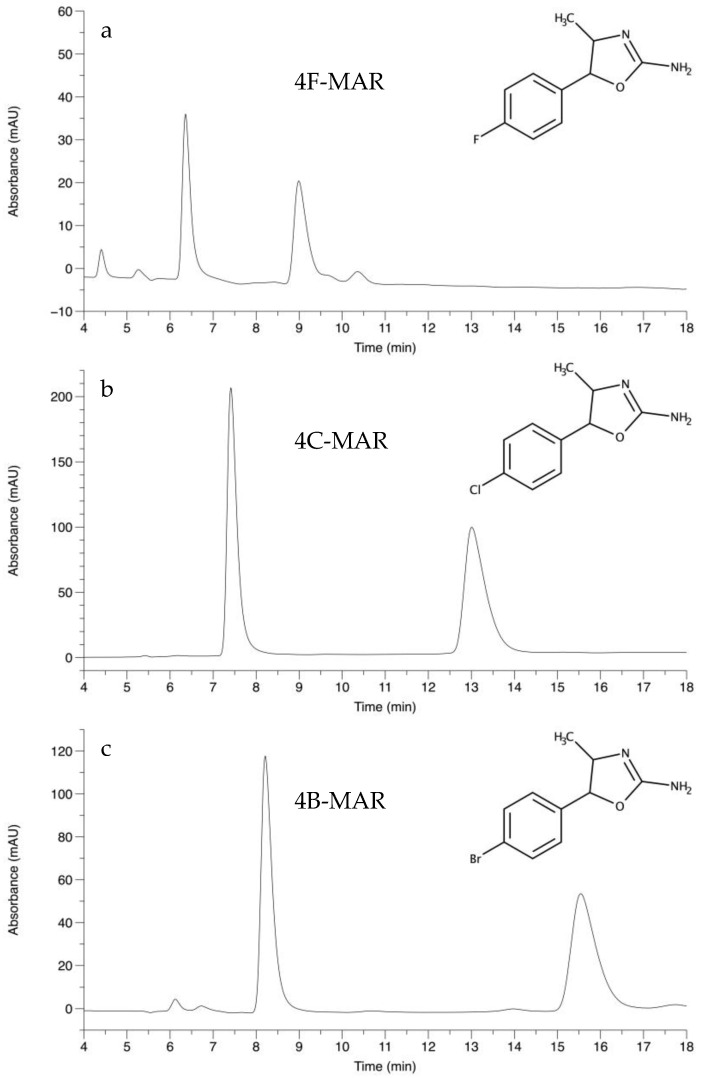
Chiral separation of (**a**) 4F-MAR, (**b**) 4C-MAR, and (**c**) 4B-MAR; Conditions: Column: Lux i-Amylose-1, 250 × 4.6 mm (5 µm), mobile phase: *n*-hexane: isopropanol: diethylamine (90:10:0.1), column temperature 25 ± 1 °C, flow rate: 2.0 mL/min, detection wavelength: 220 nm, injection volume: 5 µL.

As shown earlier, chiral HPLC columns based on derivatized polysaccharides such as amylose or cellulose are suitable for chiral separation of a broad spectrum of NPS [18,19,20]. Based on these findings, a Lux i-Amylose-1 column was chosen for the chiral separation experiments of the three 4-methylaminorex derivatives. Results of chiral HPLC combined with NMR-findings revealed that all three analytes were present as racemic mixtures of the *trans* diastereomer. The size of the respective halogen atom influenced chromatographic behavior. Increasing size of the introduced halogen atom resulted in an increase in both chromatographic resolution and retention times. Results are displayed in Table 4 and the chromatograms are shown in Figure 2. All analytes were recorded within 17 min. Ratios of peak areas were roughly 1:1.

## 3. Discussion

The investigated halogenated 4-methylaminorex derivatives possess two chiral centers leading to the possible existence of four stereoisomers, (±)-*cis* and (±)-*trans*. It was shown via NMR measurements that only the *trans*-form was present in all checked samples. For their parent compound, 4-methylaminorex, pharmacological experiments in rats have revealed differing potencies concerning stimulus properties for its four isomers, with *trans*(4*S*,5*S*) > *cis*(4S,5*R*) = *cis*(4*R*,5*S*) > *trans*(4*R*,5*R*) [21]. *Cis* (±)-4-methylaminorex appeared as a new designer drug on the drug market in 1990 under various names like “U4Euh” or “ICE” [21]. Also, for some other abused substances these enantiospecific differences could be observed and were investigated. The *S*(+)-enantiomer of amphetamine and methamphetamine possesses a higher psychoactive potency compared to the *R*(−)-form [22,23]. The *S*-enantiomer of 3,4-methylenedioxymethamphetamine (MDMA) was shown to be eliminated at a higher rate, most likely due to stereoselective metabolism [24]. For 3,4-methylenedioxypyrovalerone (MDPV) it was found that *S*(+)-MDPV is the more potent enantiomer [25]. In 2017 it was shown for mephedrone that the *R*-enantiomer is mainly responsible for the rewarding and motivational properties of the drug [26]. Due to the novelty of the halogenated MARs investigated herein, these investigations are still missing, and further studies need to be done to examine potential pharmacological and toxicological differences between the four stereoisomers.

## 4. Materials and Methods

4′-Fluoro-4-methylaminorex, 4′-chloro-4-methylaminorex, and 4′-bromo-4-methylaminorex were purchased as their hydrochloric salts from www.rarechems.com (this internet shop has since closed).

### 4.1. NMR Spectroscopy

1D proton and carbon and 2D COSY, HSQC and HMBC experiments were recorded on a 400 MHz Bruker Avance Neo spectrometer (Bruker Corporation, Billerica, Massachusetts, USA) for 4F-MAR, 4C-MAR, and 4B-MAR in DMSO-d_6_. In addition, proton and carbon spectra were recorded for the three 4-methylaminorex derivatives in CDCl_3_. Each sample consisted of approximately 5 mg of substance in 0.72 mL solvent, TMS was used as internal standard, the experimental temperature was 25 °C.

### 4.2. LC-HRMS Experimental Parameters

HR mass spectra were recorded by analyzing methanolic solutions of the compounds on an Ultimate 3000 HPLC system hyphenated to a Q Exactive Hybrid Quadrupole-Orbitrap mass spectrometer (Thermo Fisher Scientific, Waltham, MA, USA). A Poroshell 120 EC-C18 column (4.6 × 50 mm, 2.7-Micron, Agilent Technologies, Waldbronn, Germany) was used as stationary phase, and water and acetonitrile, both containing 0.1% formic acid, were used as mobile phases A and B, respectively. The gradient was as follows: 0–3 min, 20–100% B in A, 3–3.5 min 100% B, 3.5–4 min, 100–20% B in A, 4–6 min, re-equilibration. Column temperature was 25 °C and flow rate was 1 mL/min.

MS spectra were acquired in HESI positive mode; probe heater temperature was set to 350 °C, capillary temperature was 330 °C, spray voltage was 3.5 kV, sheath gas flow 65, auxiliary gas flow 20 and spare gas flow 2 arbitrary units. S-lens RF level was 50. Resolution was set to 70.000 (FWHM) for full scan, and to 17.500 for ddMS^2^. For assessment of MS/MS fragmentation patterns, measurements were performed at two different normalized collision energies, namely NCE 18 and NCE 45.

### 4.3. Chiral Chromatography

#### 4.3.1. Chemicals and Solutions

Isopropanol and and *n*-hexane were purchased from Fisher Scientific, Loughborough, UK. Diethylamine was obtained from VWR, Fontenay-sous-Bois, France. All chemicals were of analytical grade. The analytes were dissolved in isopropanol (0.5 mg/mL). To achieve complete dissolution, the samples were put into an ultrasonic bath for five minutes.

#### 4.3.2. Chromatographic Conditions

HPLC-UV measurements were carried out by an Agilent 1260 Series Infinity II Liquid Chromatograph (Agilent Technologies, Waldbronn, Germany) equipped with an autosampler and a variable wavelength detector. UV-data were collected at 220 nm. OpenLAB Chromatography Data System (CDS), ChemStation Edition for LC and LC/MS Systems Rev. C. 01.07SR2[255] by Agilent Technologies (Waldbronn, Germany) served for data processing. A commercially available Lux i-Amylose-1 column by Phenomenex (Aschaffenburg, Germany) with a length of 250 mm, a diameter of 4.6 mm, and a particle size of 5 µm was used as stationary phase. Column temperature was kept constant at 25 ± 1 °C. The mobile phase was prepared by mixing *n*-hexane and isopropanol (IPA) in a ratio of 90:10 (*v*/*v*). Diethylamine (DEA) was added as basic modifier for a final mobile phase composition of *n*-hexane: IPA: DEA = 90:10:0.1. Flow rate was 2 mL/min and injection volume was 5 µL. Measurements were performed under isocratic conditions.

## 5. Conclusions

Three novel MARs, recently available via the internet have been introduced and characterized by means of NMR, HR-MS as well as chiral chromatography. NMR measurements revealed that only the *trans*-form was present in all three samples and chiral HPLC experiments showed that the compounds were traded as racemic mixtures. LC-HRMS measurements were in accordance with the structures suggested from NMR measurements and isotopic patterns showed the existence of the three halogen atoms claimed by the provider.

To date, no pharmacological or toxicological studies are available for these compounds and further studies need to be done to elucidate the potency of the four stereoisomers. For this reason, the consumption of the tested compounds is problematic and risky.

## Figures and Tables

**Table 1:** ^1^H and ^13^C chemical shift values (δ, ppm) of the investigated 4-methylaminorex derivatives in CDCl_3_ at 25 °C with TMS as internal standard, *J*-values in Hz.

Position	4B-MAR		4C-MAR		4F-MAR	
	δ_C_	δ_H_	δ_C_	δ_H_	δ_C_	δ_H_
2	159.3	-	159.1	-	158.9	-
4	68.6	3.87 (p, 6.4)	68.7	3.88 (p, 6.9)	68.7	3.91 (p, 6.8)
5	88.2	4.87 (d, 6.8)	88.2	4.88 (d, 7.2)	88.5	4.90 (d, 7.2)
2-NH_2_	-	4.82 (brs)	-	4.54 (brs)	-	4.34 (brs)
4-CH_3_	21.6	1.32 (d, 7.1)	21.6	1.32 (d, 6.4)	21.6	1.32 (d, 6.6)
1′	139.1	-	138.5	-	135.7 (d, 3.2)	-
2′/6′	127.3	7.20 (d, 8.5)	127.1	7.26 (d, 8.6)	127.6 (d, 8.3)	7.31 (dd, 8.8, 5.3)
3′/5′	131.9	7.50 (d, 8.5)	128.9	7.34 (d, 8.6)	115.7 (d, 21.6)	7.03 (t, 8.8)
4′	122.2	-	134.1	-	162.7 (d, 246.8)	-

**Table 2:** ^1^H and ^13^C chemical shift values (δ, ppm) of the investigated 4-methylaminorex derivatives in DMSO-d_6_ at 25 °C with TMS as internal standard, *J*-values in Hz.

Position	4B-MAR		4C-MAR		4F-MAR	
	δ_C_	δ_H_	δ_C_	δ_H_	δ_C_	δ_H_
2	158.8	-	158.8	-	158.8	-
4	68.5	3.61 (p, 6.4)	68.5	3.62 (p, 6.3)	68.4	3.64 (p, 6.5)
5	85.3	4.86 (d, 6.4)	85.3	4.88 (d, 6.3)	85.5	4.87 (d, 6.5)
2-NH_2_	-	5.97 (s)	-	5.99 (s)	-	5.96 (s)
4-CH_3_	22.0	1.17 (d, 6.4)	22.0	1.17 (d, 6.3)	22.0	1.16 (d, 6.5)
1′	140.7	-	140.2	-	137.4 (d, 3.1)	-
2′/6′	127.4	7.26 (d, 8.5)	127.1	7.33 (d, 8.4)	127.4 (d, 8.3)	7.35 (dd, 8.9, 5.4)
3′/5′	131.4	7.58 (d, 8.5)	128.5	7.45 (d, 8.4)	115.3 (d, 21.4)	7.20 (t, 8.9)
4′	120.7	-	132.2	-	161.6 (d, 243.5)	-

**Table 3:** LC-HRMS data of 4F-MAR, 4C-MAR and 4B-MAR in the HESI positive ion mode.

Compound	Found *m*/*z* [Ion Species]	Molecular Formula (Ion Species)	*m*/*z* Calculated for Molecular Formula	Δ (ppm)
4F-MAR	195.0929 [M + H]^+^	C_10_H_12_ON_2_F^+^	195.0934	0.63
4C-MAR	211.0634 [M + H]^+^	C_10_H_12_ON_2_Cl^+^	211.0638	0.72
4B-MAR	255.0129 [M + H]^+^	C_10_H_12_ON_2_Br^+^	255.0133	0.66

**Table 4:** LC-HRMS Chiral separation results of the three 4-methylaminorex derivatives. Retention times (t) of both enantiomers, separation factors (α) and chromatographic resolution (R_s_) are given. Conditions: Column: Lux i-Amylose-1, 250 × 4.6 mm (5 µm), mobile phase: *n*-hexane: isopropanol: diethylamine (90:10:0.1), column temperature 25 ± 1 °C, flow rate: 2.0 mL/min, detection wavelength: 220 nm, injection volume: 5 µL.

Compound	t_1_	t_2_	α	R_s_
4F-MAR	6.36	8.99	1.53	9.49
4C-MAR	7.41	13.01	1.93	14.70
4B-MAR	8.21	15.54	2.08	16.41

## Data Availability

Not applicable.

## Supplementary Materials

The following supporting information can be downloaded at: https://www.mdpi.com/article/10.3390/molecules27185770/s1, Figure S1: Proton NMR spectrum (400 MHz, CDCl_3_) 4B-MAR; Figure S2: Carbon NMR spectrum (100 MHz, CDCl_3_) 4B-MAR; Figure S3: Proton NMR spectrum (400 MHz, CDCl_3_) 4C-MAR; Figure S4: Carbon NMR spectrum (100 MHz, CDCl_3_) 4C-MAR; Figure S5: Proton NMR spectrum (400 MHz, CDCl_3_) 4F-MAR; Figure S6: Carbon NMR spectrum (100 MHz, CDCl_3_) 4F-MAR; Figure S7: Proton NMR spectrum (400 MHz, DMSO-d_6_) 4B-MAR; Figure S8: Carbon NMR spectrum (100 MHz, DMSO-d_6_) 4B-MAR; Figure S9: DQF-COSY NMR spectrum (400 MHz, DMSO-d_6_) 4B-MAR; Figure S10: HSQC NMR spectrum (100/400 MHz, DMSO-d_63_) 4B-MAR; Figure S11: HMBC NMR spectrum (100/400 MHz, DMSO-d_6_) 4B-MAR; Figure S12: Proton NMR spectrum (400 MHz, DMSO-d_6_) 4C-MAR; Figure S13: Carbon NMR spectrum (100 MHz, DMSO-d_6_) 4C-MAR; Figure S14: DQF-COSY NMR spectrum (400 MHz, DMSO-d_6_) 4C-MAR; Figure S15: HSQC NMR spectrum (100/400 MHz, DMSO-d_63_) 4C-MAR; Figure S16: HMBC NMR spectrum (100/400 MHz, DMSO-d_6_) 4C-MAR; Figure S17: Proton NMR spectrum (400 MHz, DMSO-d6) 4F-MAR; Figure S18: Carbon NMR spectrum (100 MHz, DMSO-d_6_) 4F-MAR; Figure S19: DQF-COSY NMR spectrum (400 MHz, DMSO-d_6_) 4F-MAR; Figure S20: HSQC NMR spectrum (100/400 MHz, DMSO-d_63_) 4F-MAR; Figure S21: HMBC NMR spectrum (100/400 MHz, DMSO-d_63_) 4F-MAR; Figure S22: ^19^F NMR spectrum (377 MHz, DMSO-d_63_) 4F-MAR; Figure S23: HESI positive mode full MS spectrum of 4B-MAR; Figure S24: HESI positive mode full MS spectrum of 4C-MAR; Figure S25: HESI positive mode full MS spectrum of 4F-MAR; Figure S26: HESI positive mode MS/MS spectrum of 4B-MAR; Figure S27: HESI positive mode MS/MS spectrum of 4C-MAR; Figure S28: HESI positive mode MS/MS spectrum of 4F-MAR.

## Sample Availability

Samples of the compounds are not available from the authors.
