# Supplementary material for: Characterization of Three Novel 4-Methylaminorex Derivatives Applied as Designer Drugs
Source: Molecules. 2022 Sep 6;27(18):5770. doi: 10.3390/molecules27185770 (PMC9503756; doi:10.3390/molecules27185770)
Supplement: Supplementary file 1 [file molecules-27-05770-s001.zip › molecules-1864273-supplementary.pdf]

molecules

Article

Characterization of Three Novel 4-Methylaminorex Derivatives Applied as Designer Drugs

Elisabeth Seibert [1], Olaf Kunert [1], Eva-Maria Pferschy-Wenzig [2] and Martin G. Schmid [1,*]

[1] Department of Pharmaceutical Chemistry, Institute of Pharmaceutical Sciences, University of Graz, Schubertstraße 1, 8010 Graz, Austria
[2] Department of Pharmacognosy, Institute of Pharmaceutical Sciences, University of Graz, Beethovenstraße 8, 8010 Graz, Austria
* Correspondence: martin.schmid@uni-graz.at

1. Supplementary materials

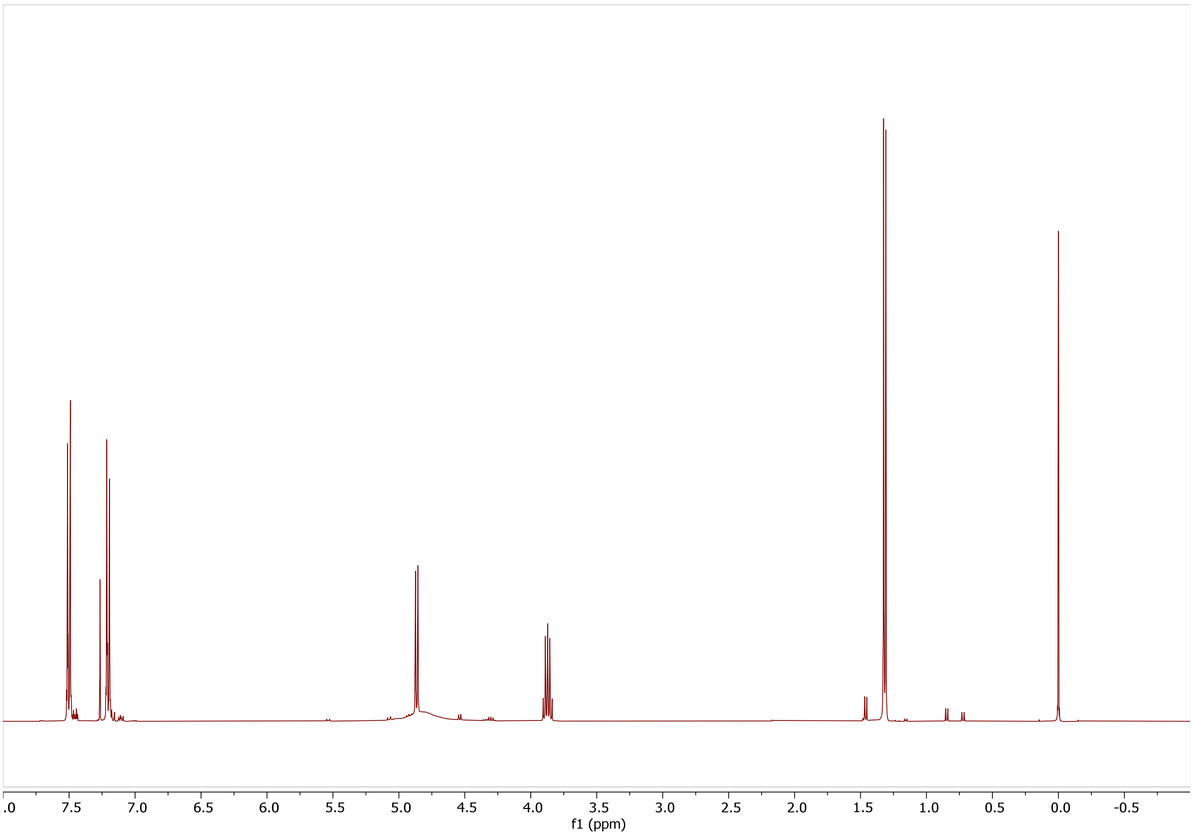

Figure S1. Proton NMR spectrum (400 MHz, $CDCl_3$) 4B-MAR.

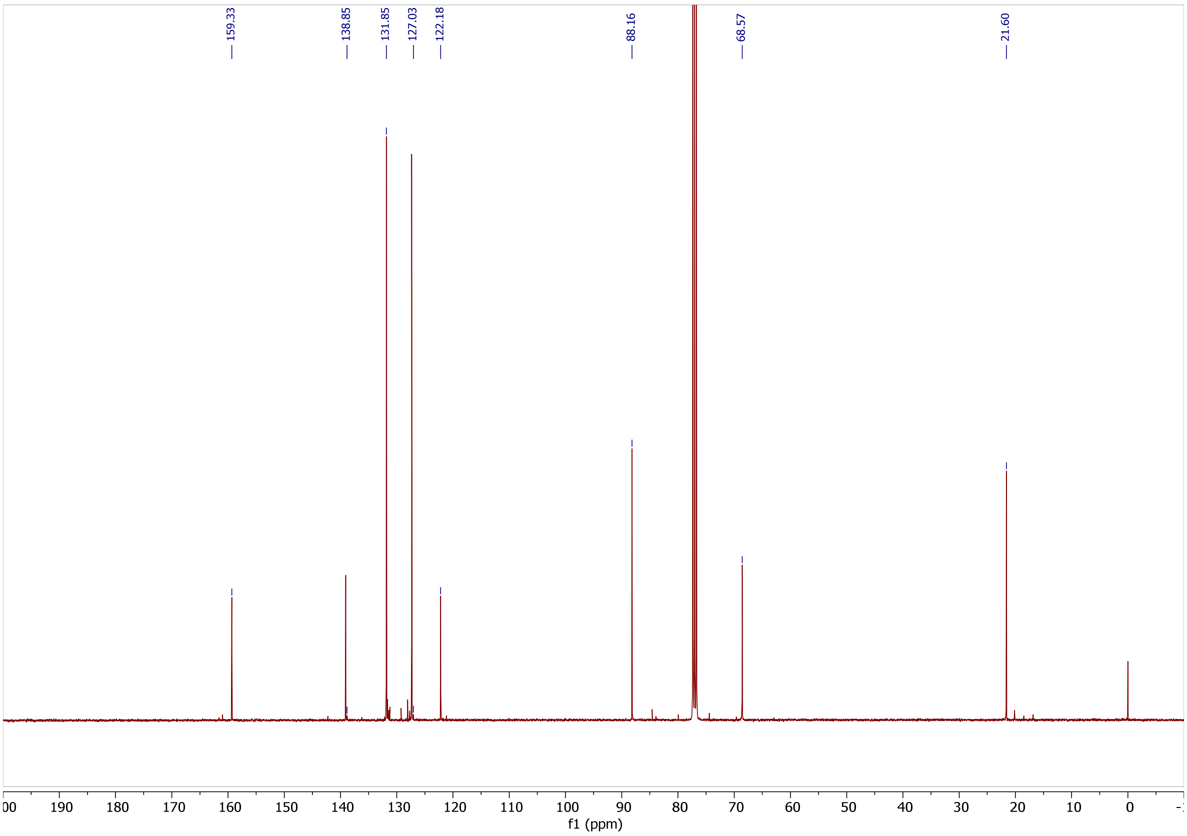

Figure S2. Carbon NMR spectrum (100 MHz, $CDCl_3$) 4B-MAR.

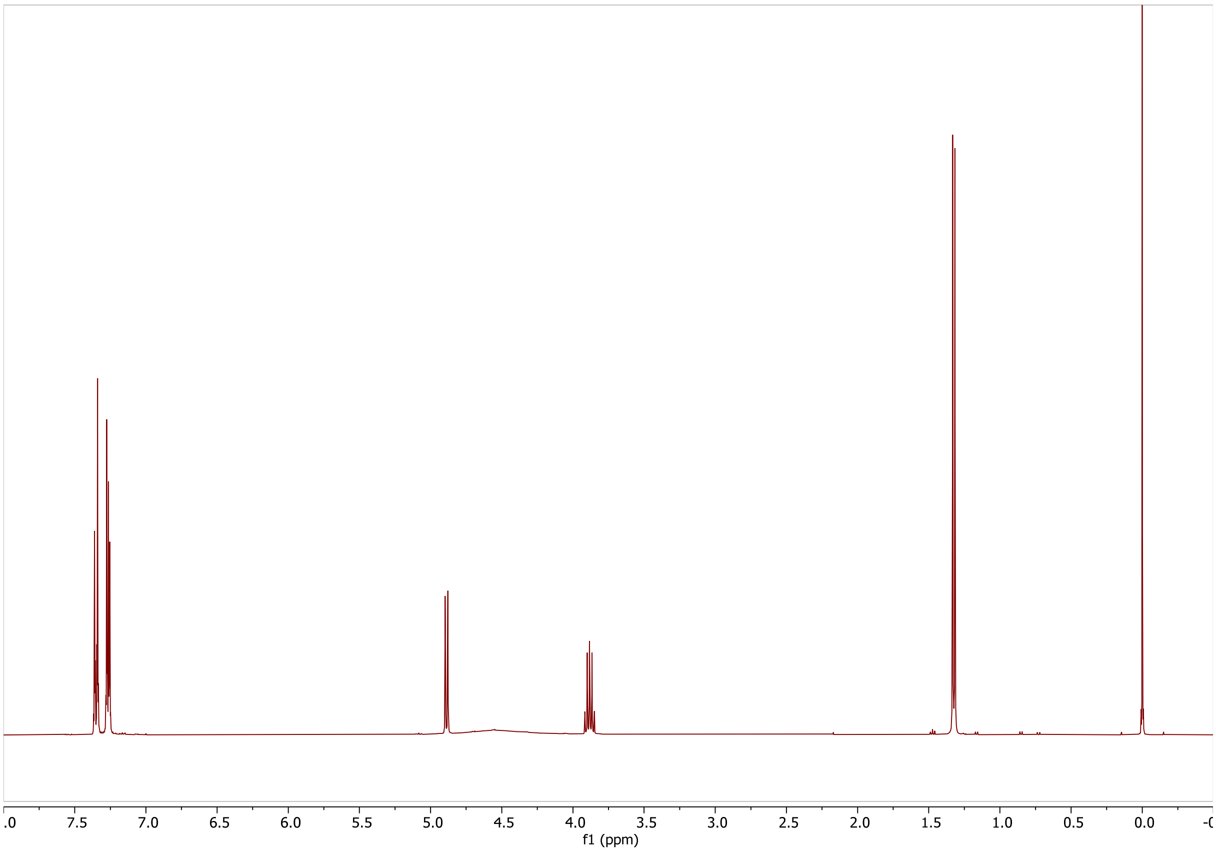

Figure S3. Proton NMR spectrum (400 MHz, $CDCl_3$) 4C-MAR.

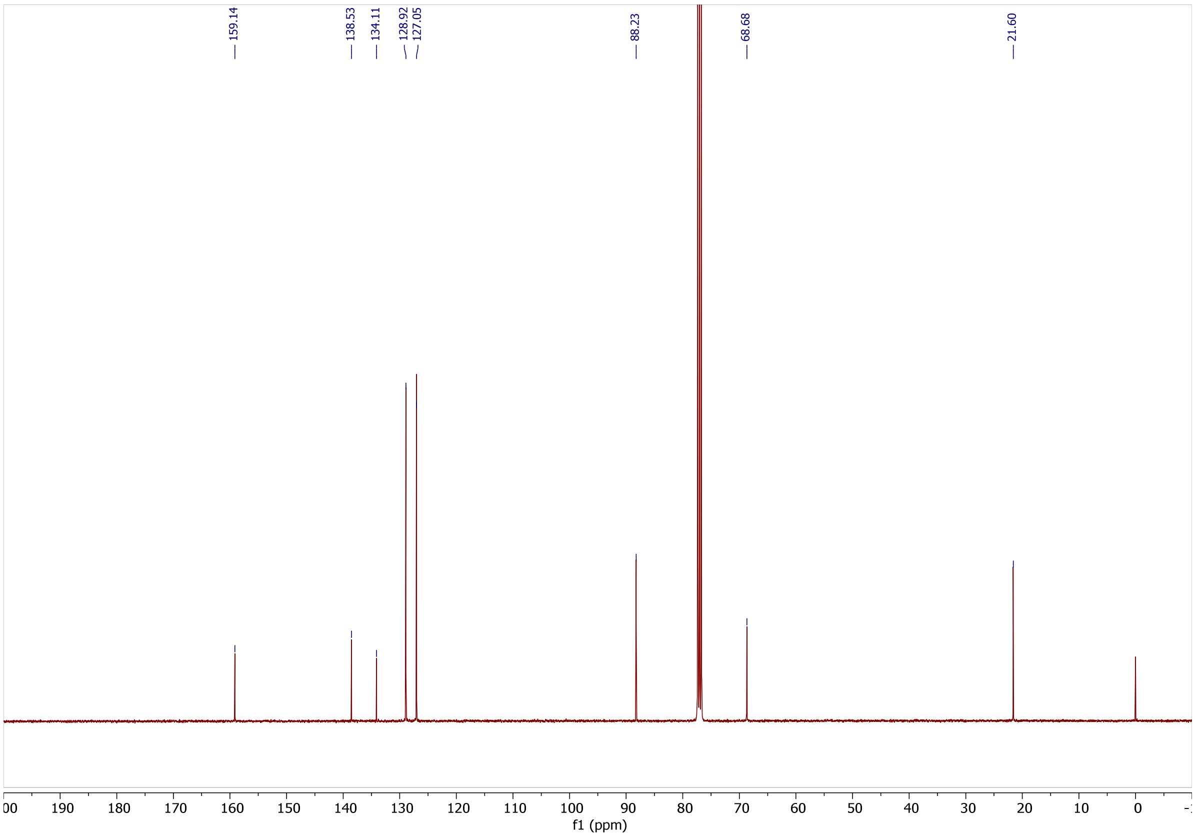

Figure S4. Carbon NMR spectrum (100 MHz, $CDCl_3$) 4C-MAR.

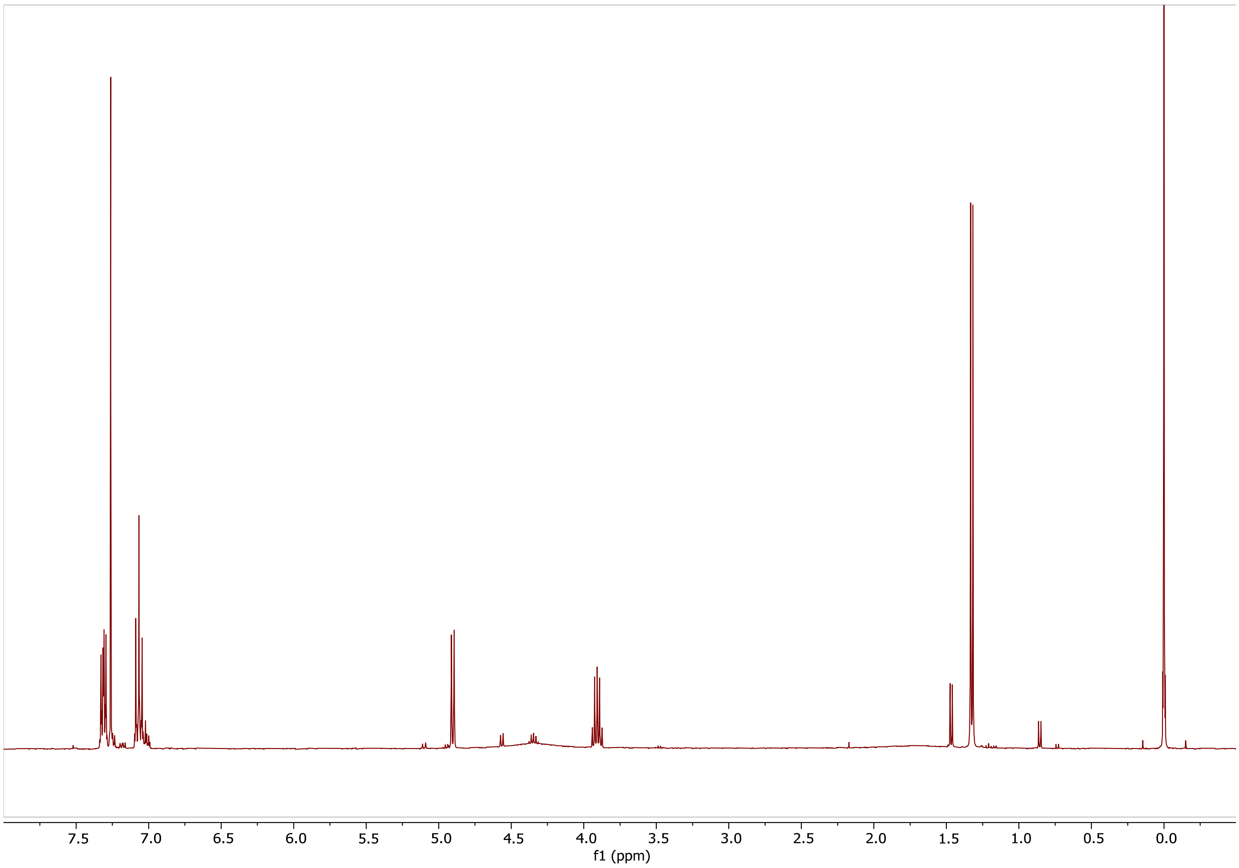

Figure S5. Proton NMR spectrum (400 MHz, $CDCl_3$) 4F-MAR.

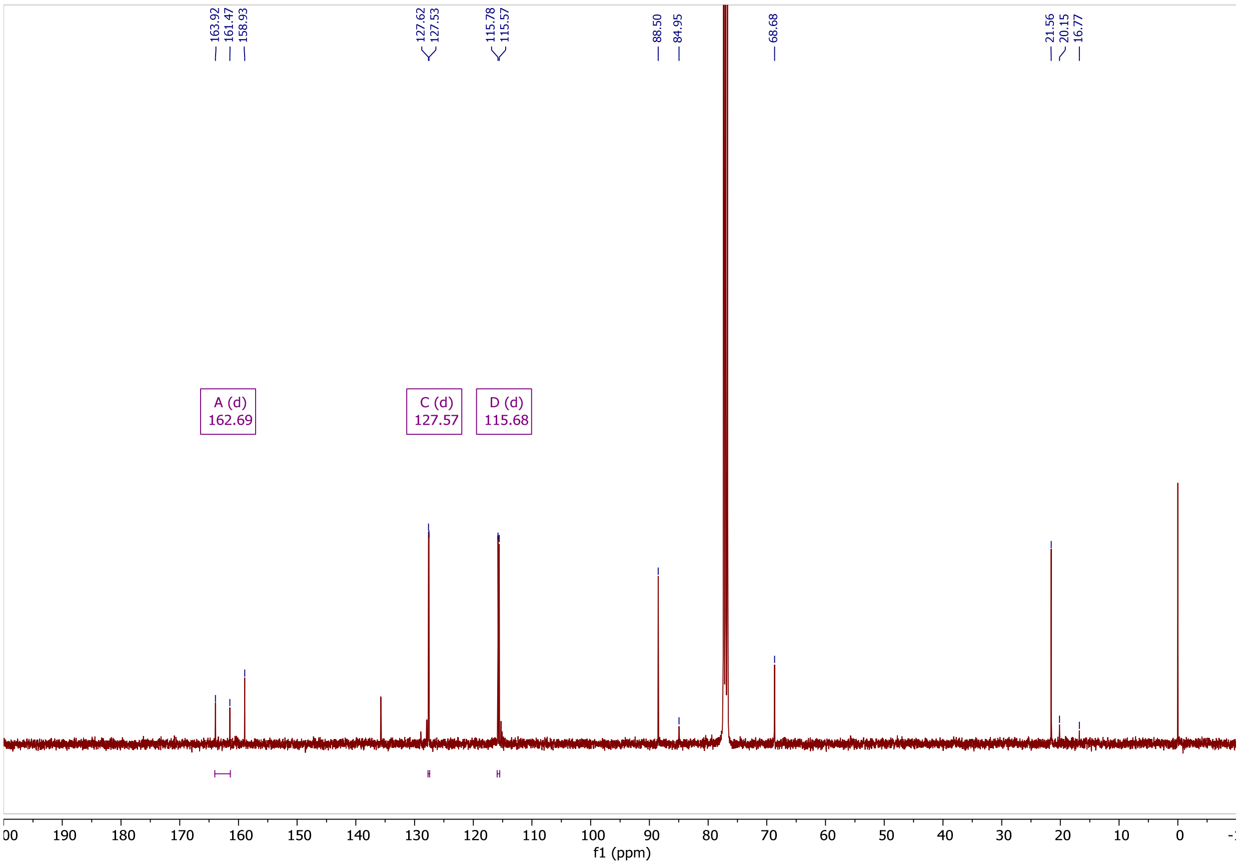

Figure S6. Carbon NMR spectrum (100 MHz, $CDCl_3$) 4F-MAR. The centers of the doublets caused by heteronuclear $^{19}F,^{13}C$-*J*-couplings are given in the boxes.

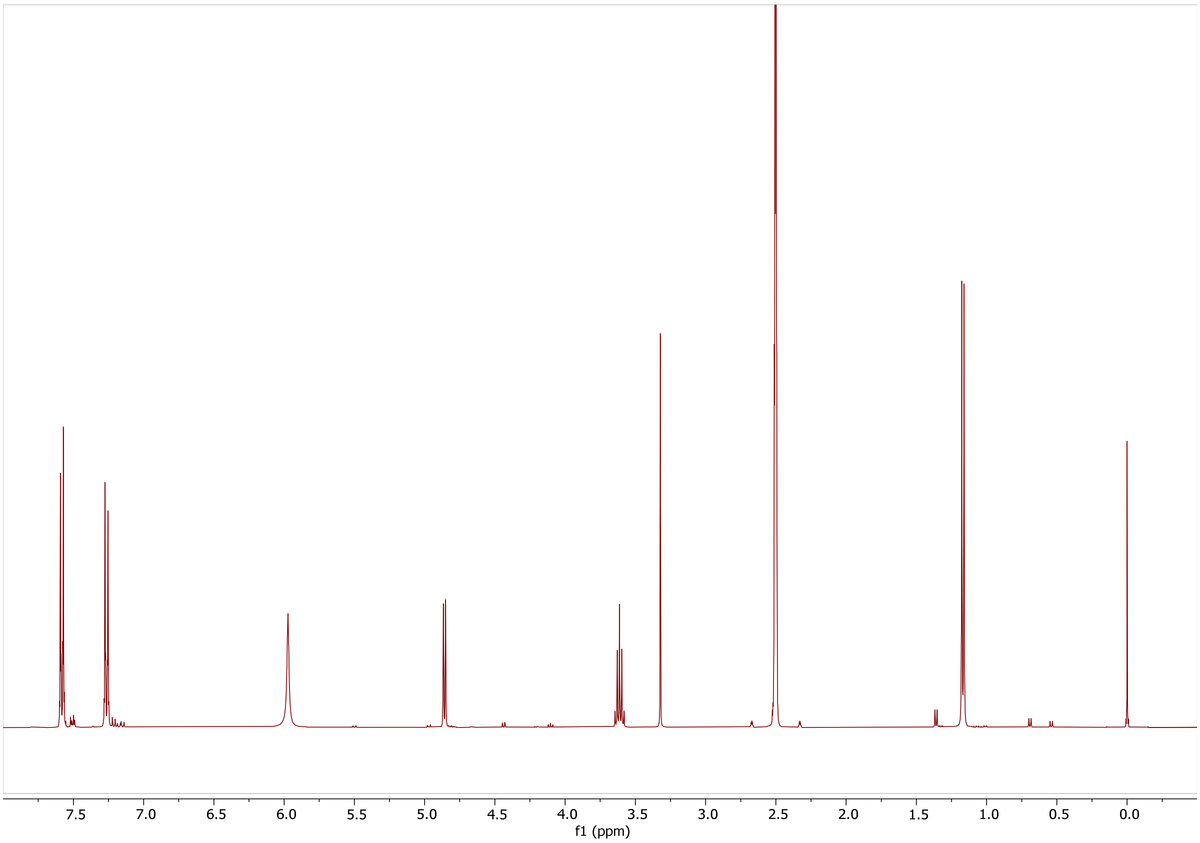

Figure S7. Proton NMR spectrum (400 MHz, DMSO-d$_6$) 4B-MAR.

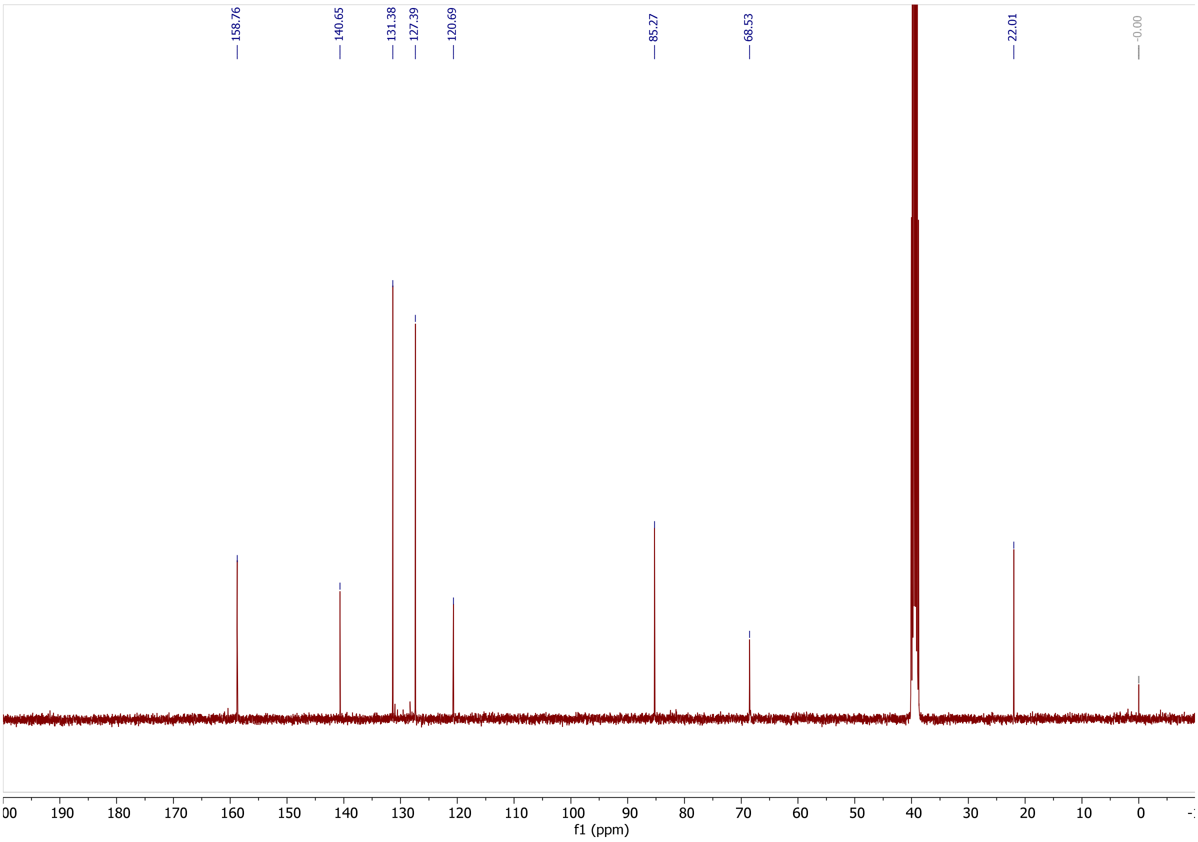

Figure S8. Carbon NMR spectrum (100 MHz, DMSO-d$_6$) 4B-MAR.

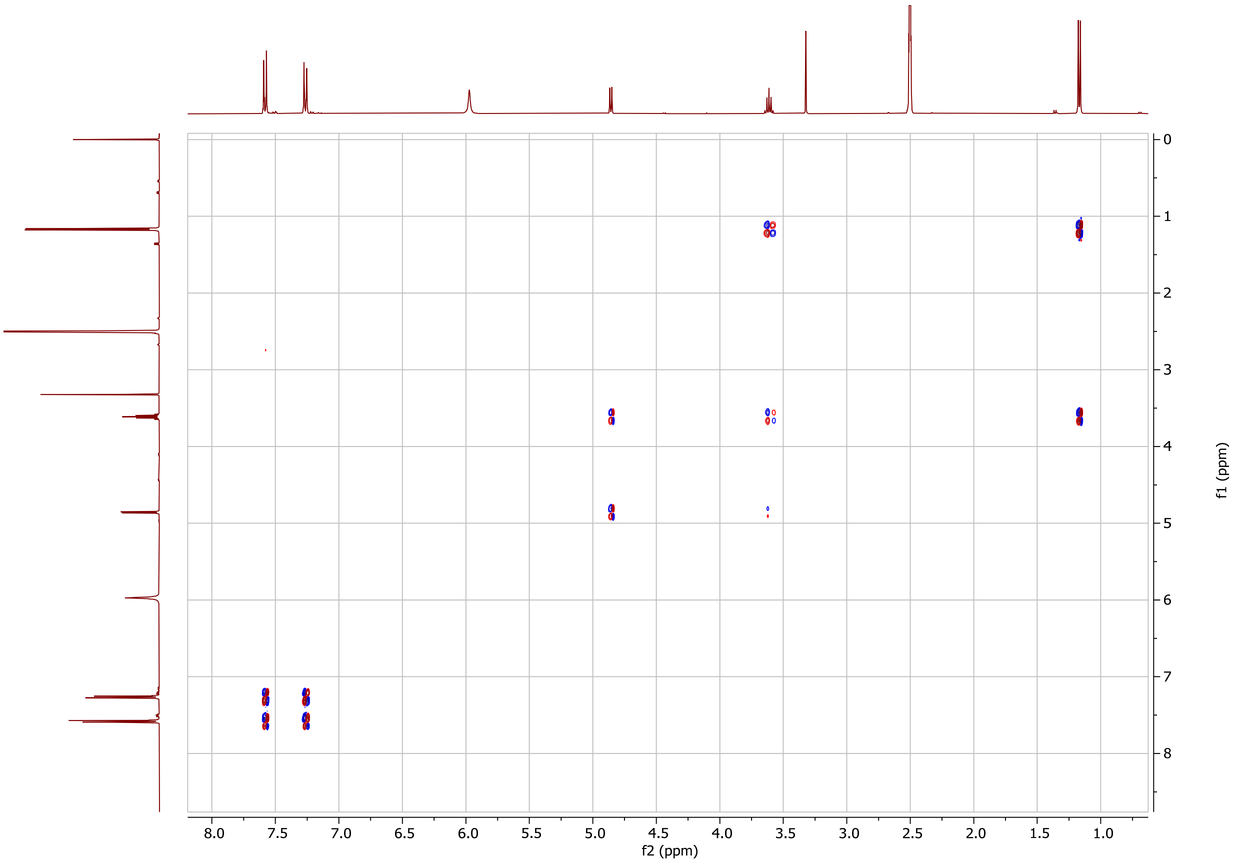

Figure S9. DQF-COSY NMR spectrum (400 MHz, DMSO-d6) 4B-MAR.

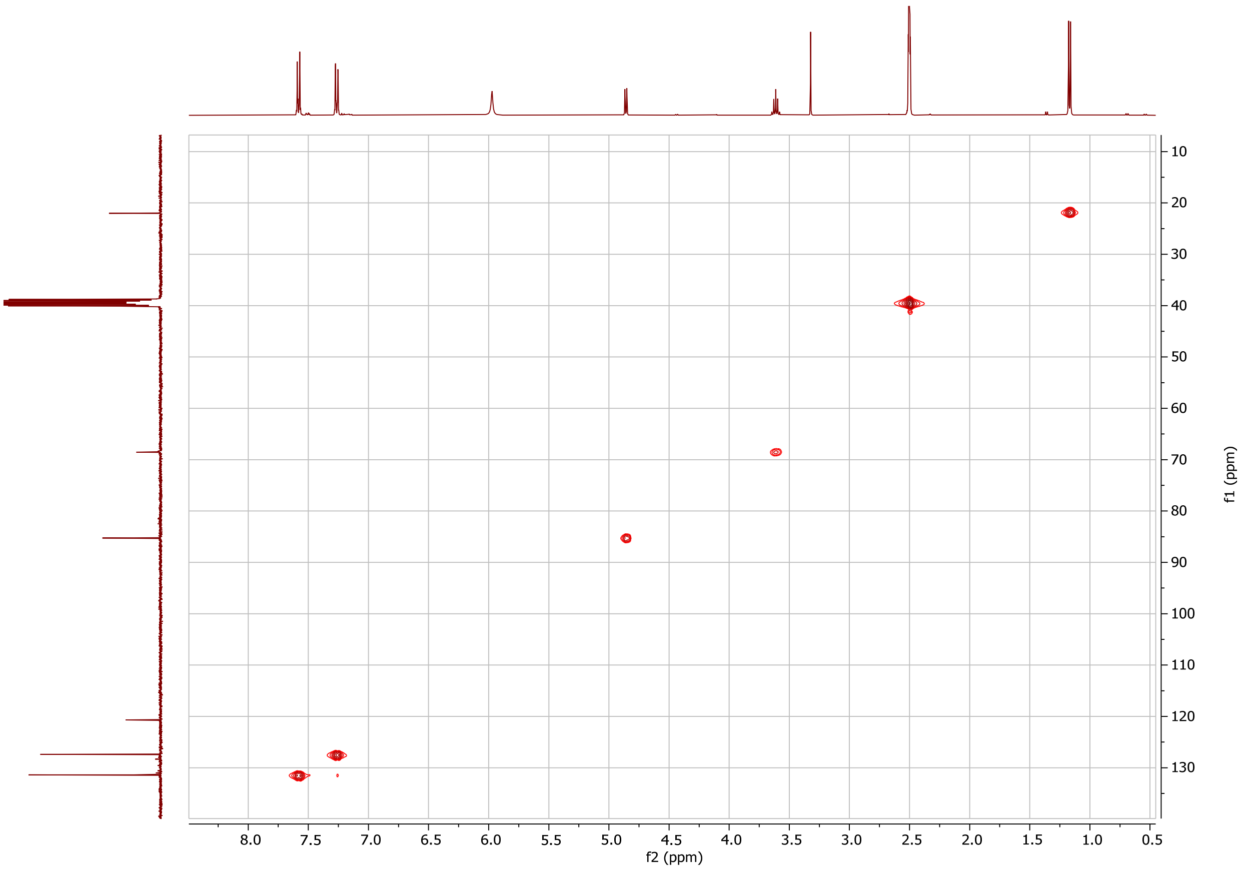

Figure S10. HSQC NMR spectrum (100/400 MHz, DMSO-d63) 4B-MAR.

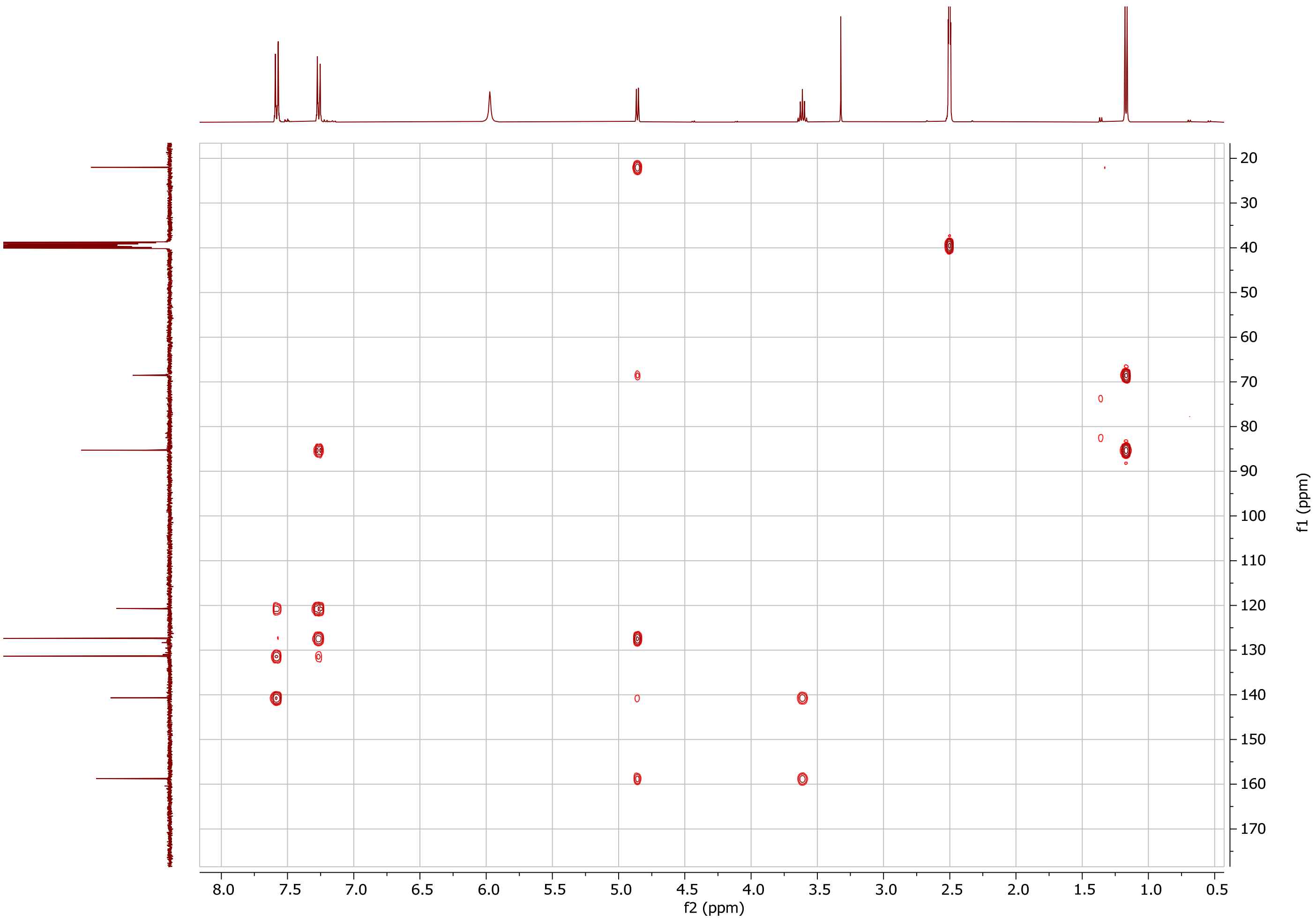

Figure S11. HMBC NMR spectrum (100/400 MHz, DMSO-d6) 4B-MAR.

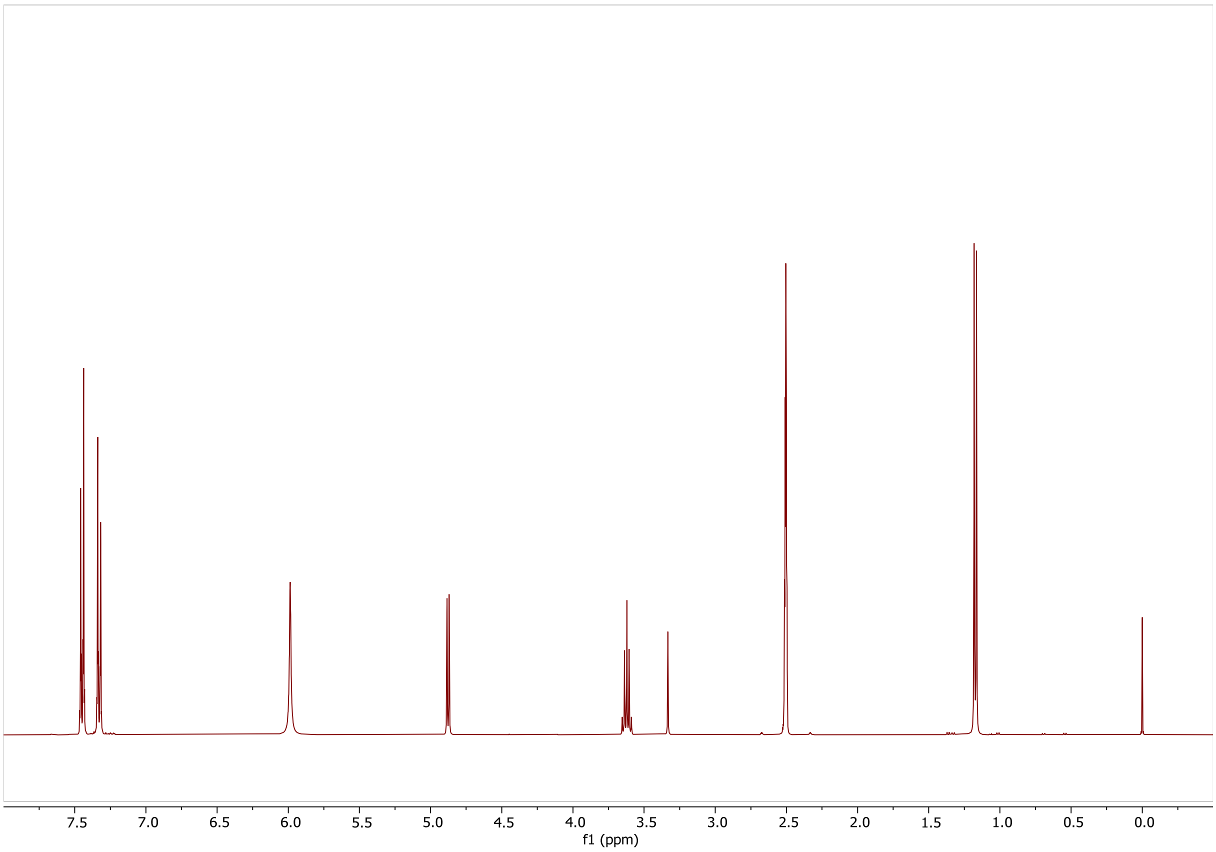

Figure S12. Proton NMR spectrum (400 MHz, DMSO-d6) 4C-MAR.

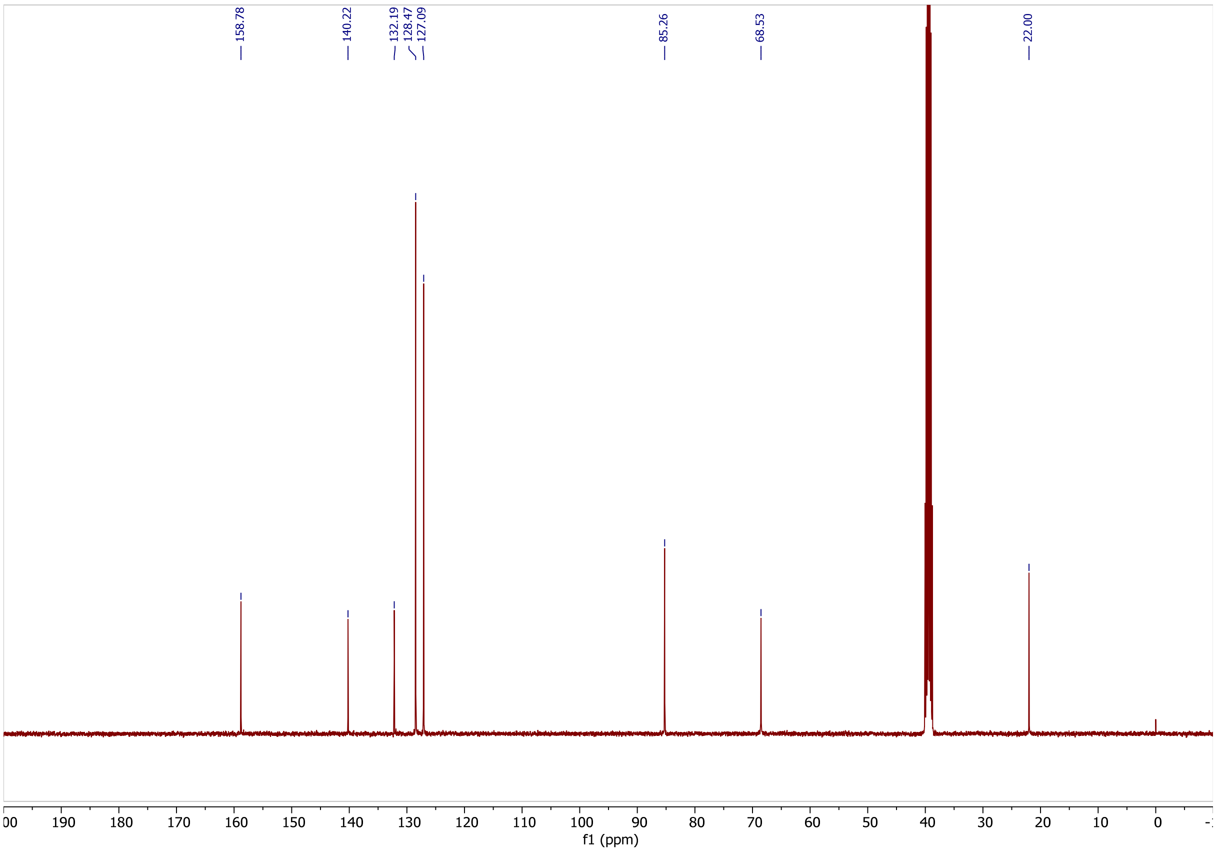

Figure S13. Carbon NMR spectrum (100 MHz, DMSO-d6) 4C-MAR.

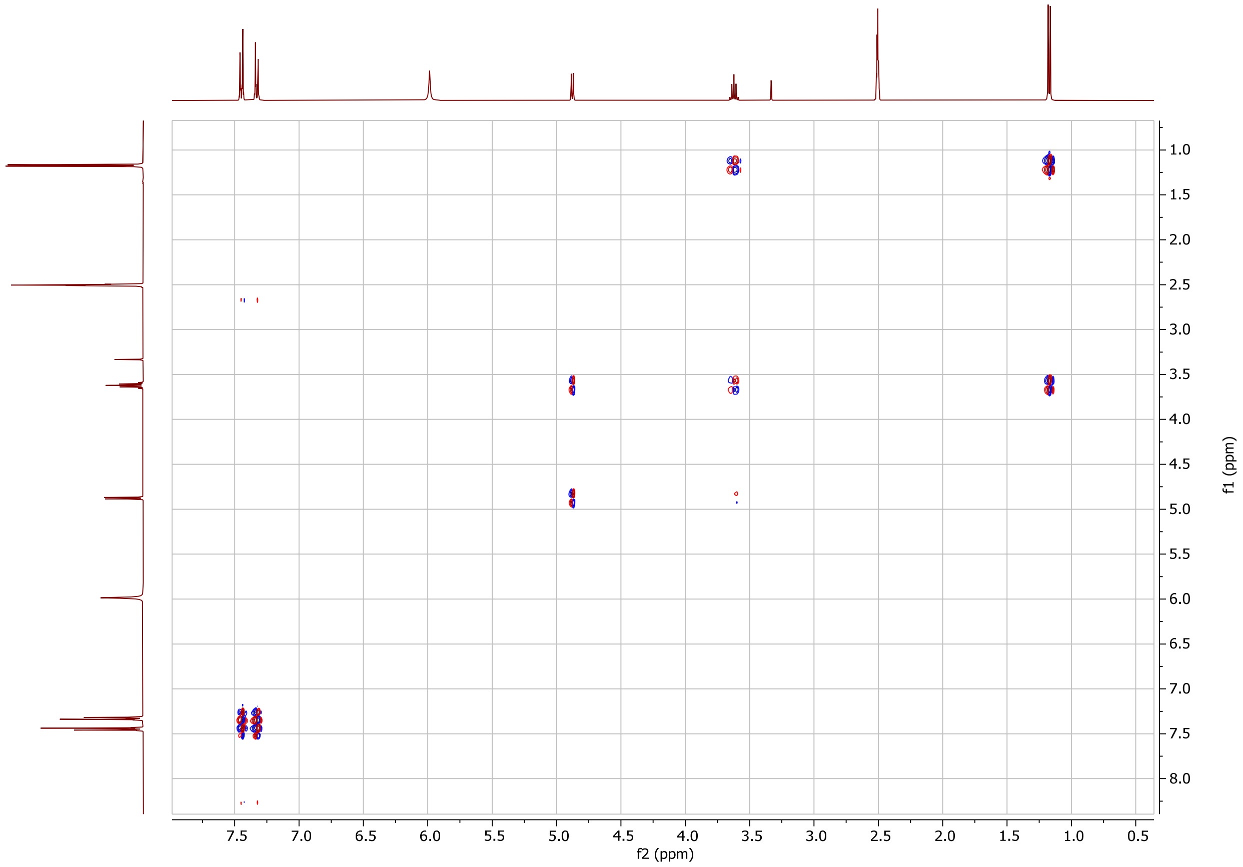

Figure S14. DQF-COSY NMR spectrum (400 MHz, DMSO-d6) 4C-MAR.

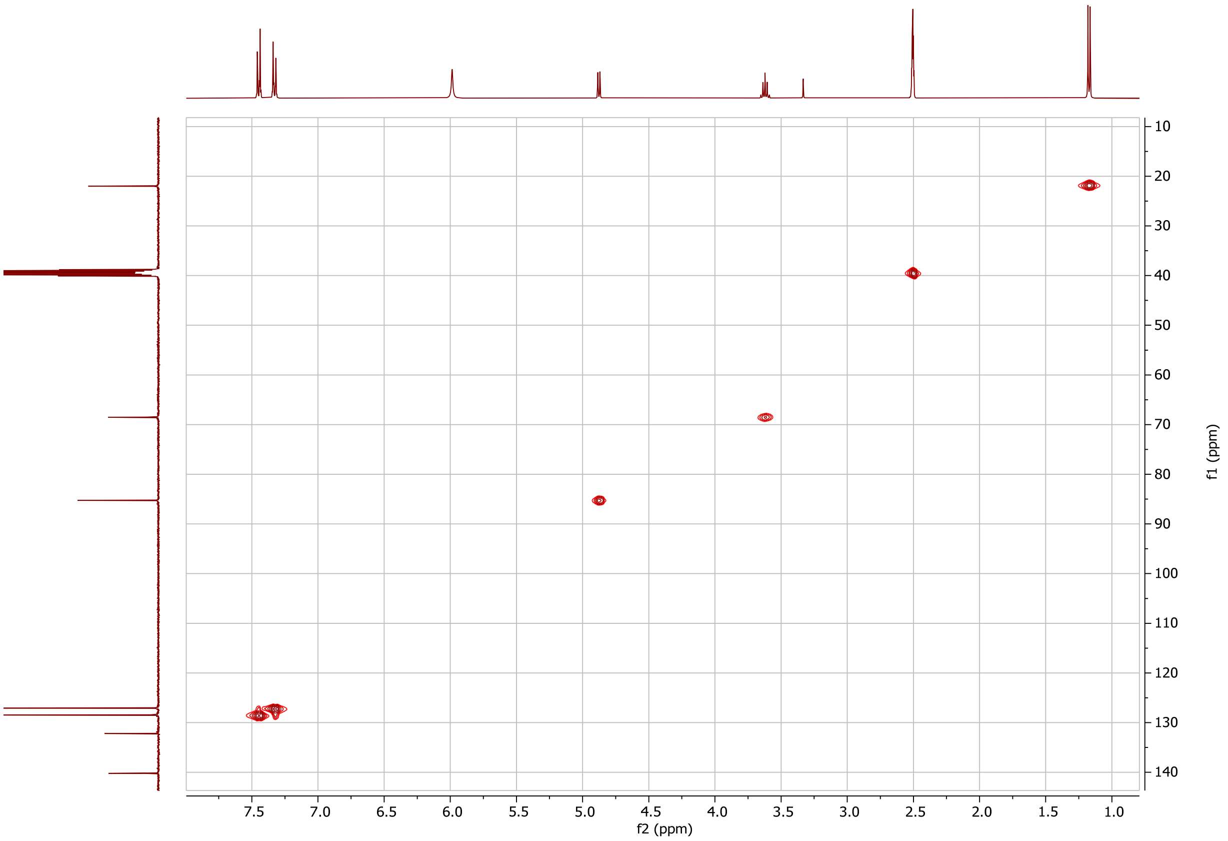

Figure S15. HSQC NMR spectrum (100/400 MHz, DMSO-d63) 4C-MAR.

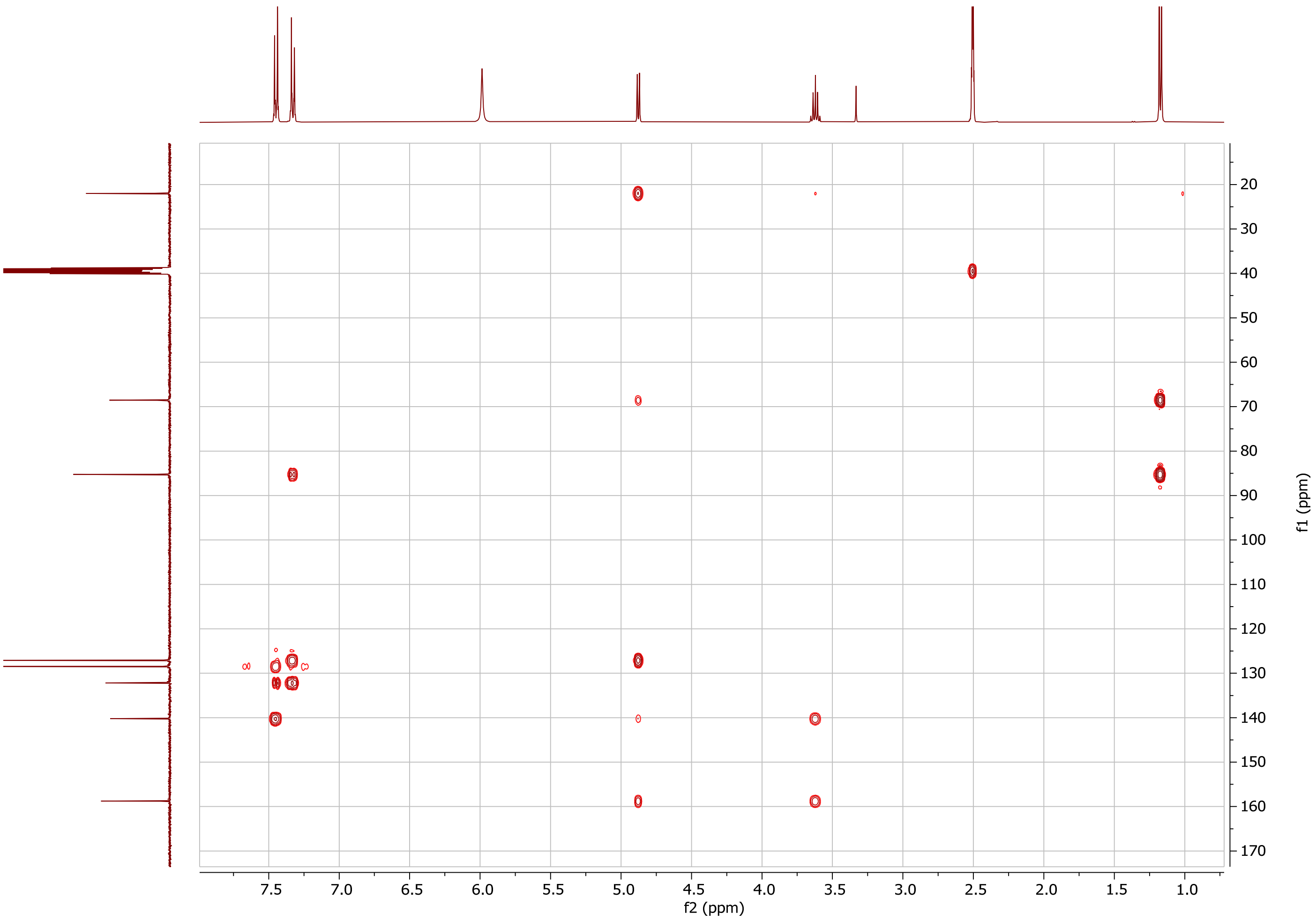

Figure S16. HMBC NMR spectrum (100/400 MHz, DMSO-d6) 4C-MAR.

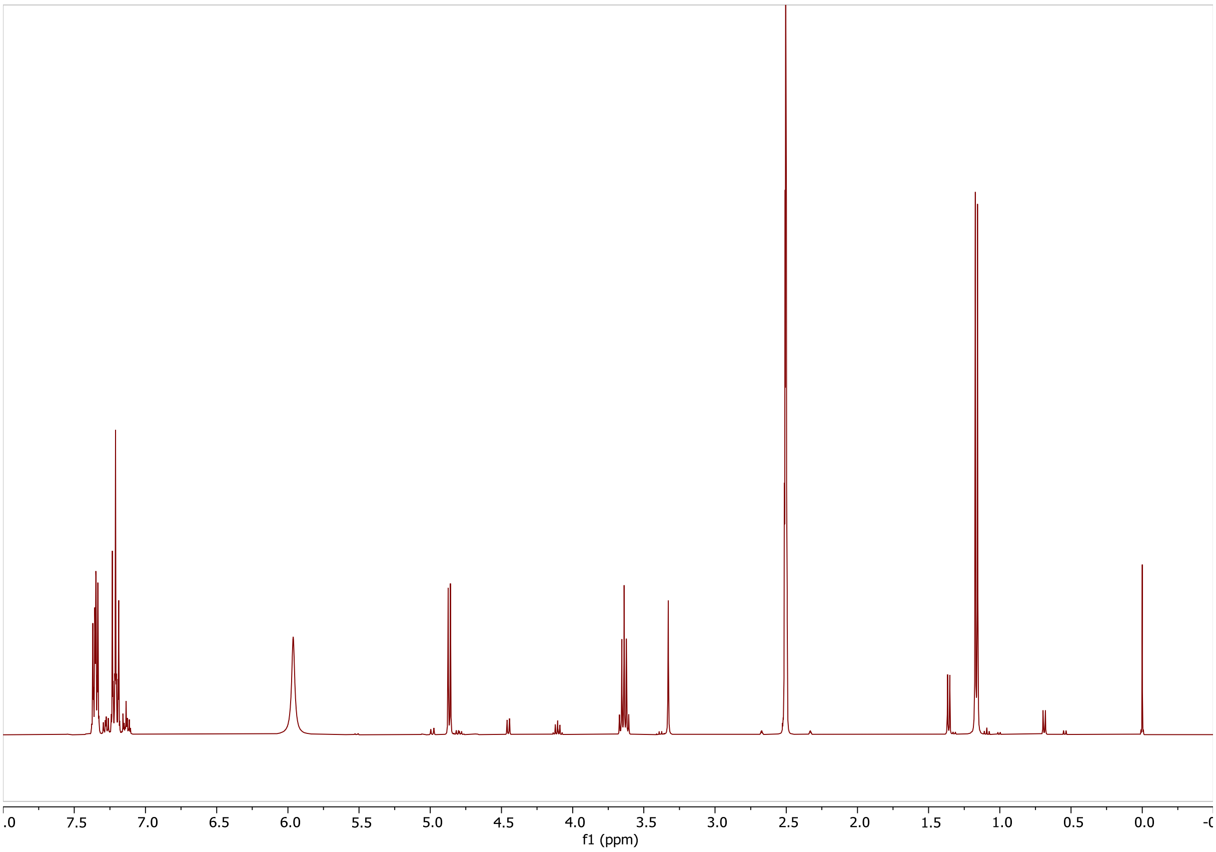

Figure S17. Proton NMR spectrum (400 MHz, DMSO-d6) 4F-MAR.

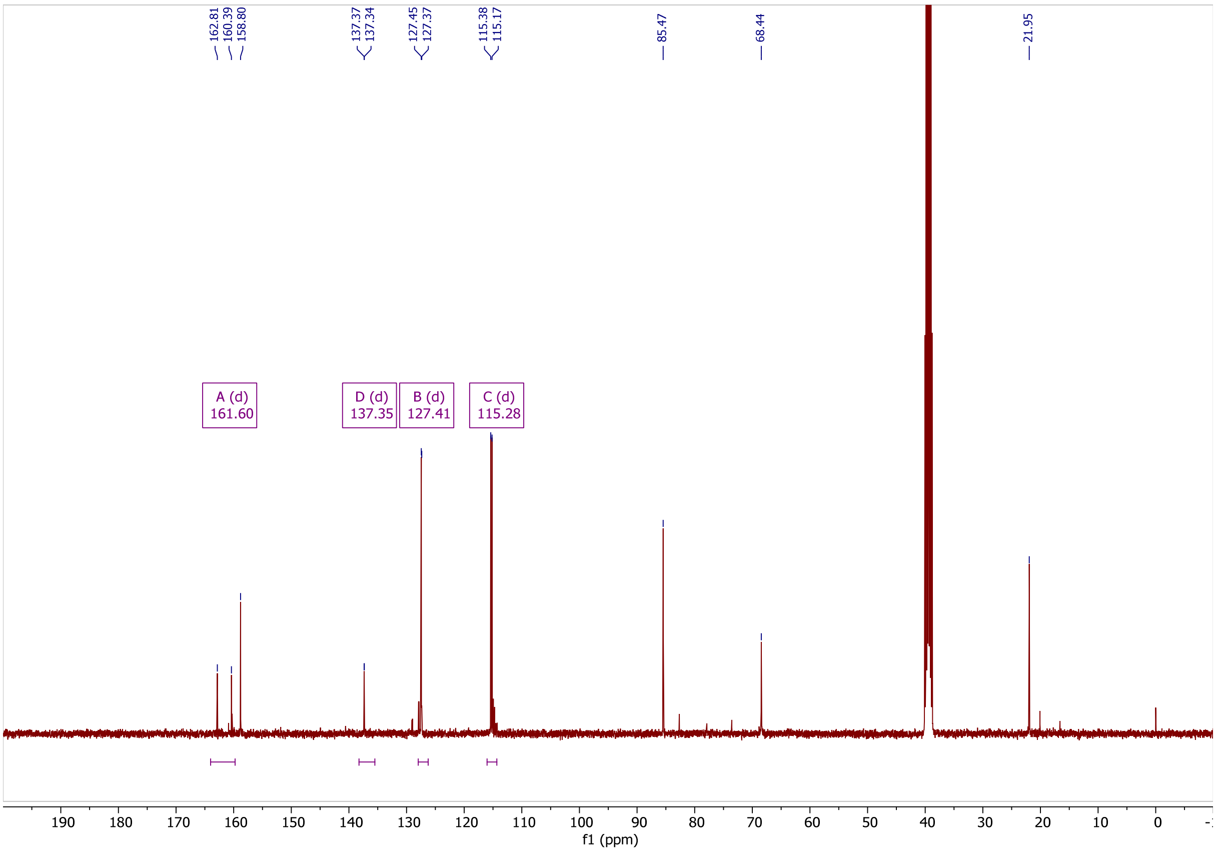

Figure S18. Carbon NMR spectrum (100 MHz, DMSO-d6) 4F-MAR. The centers of the doublets caused by heteronuclear $^{19}F,^{13}C$-*J*-couplings are given in the boxes.

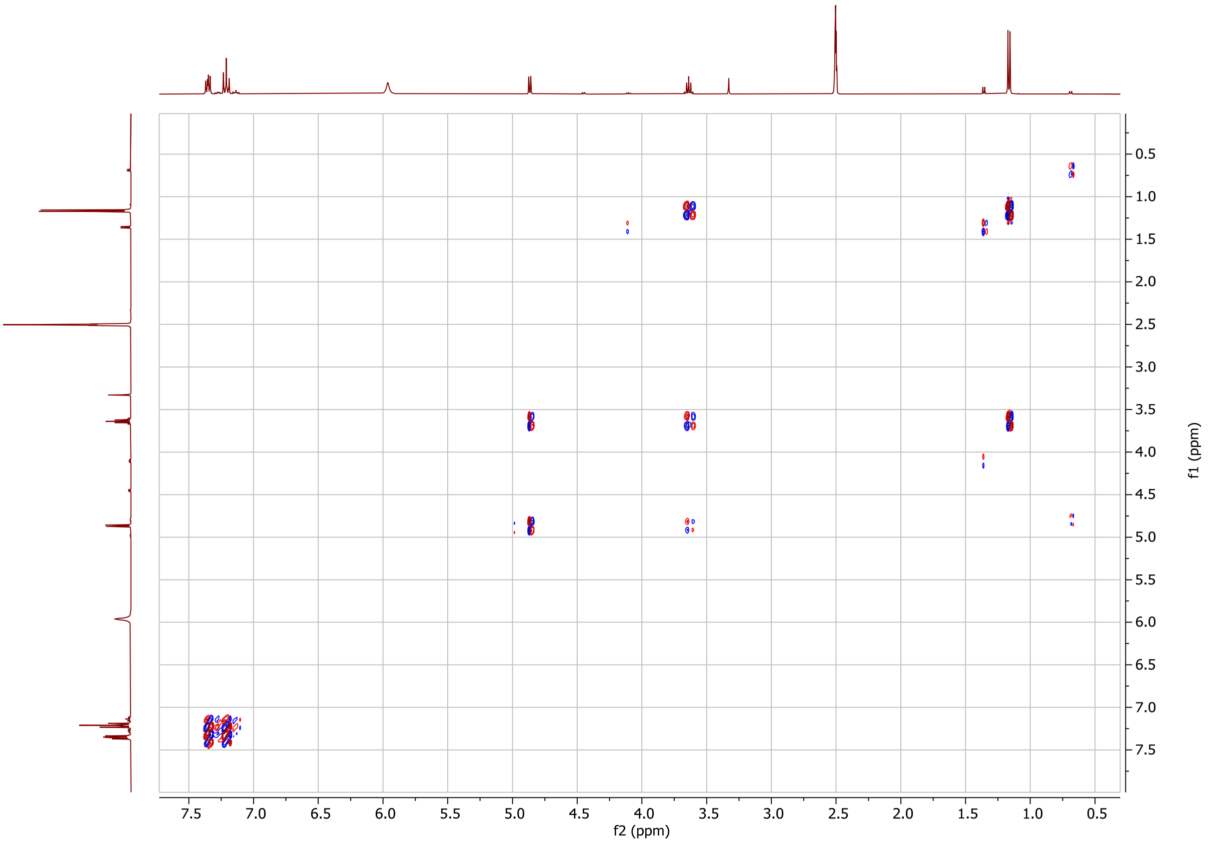

Figure S19. DQF-COSY NMR spectrum (400 MHz, DMSO-d_6) 4F-MAR.

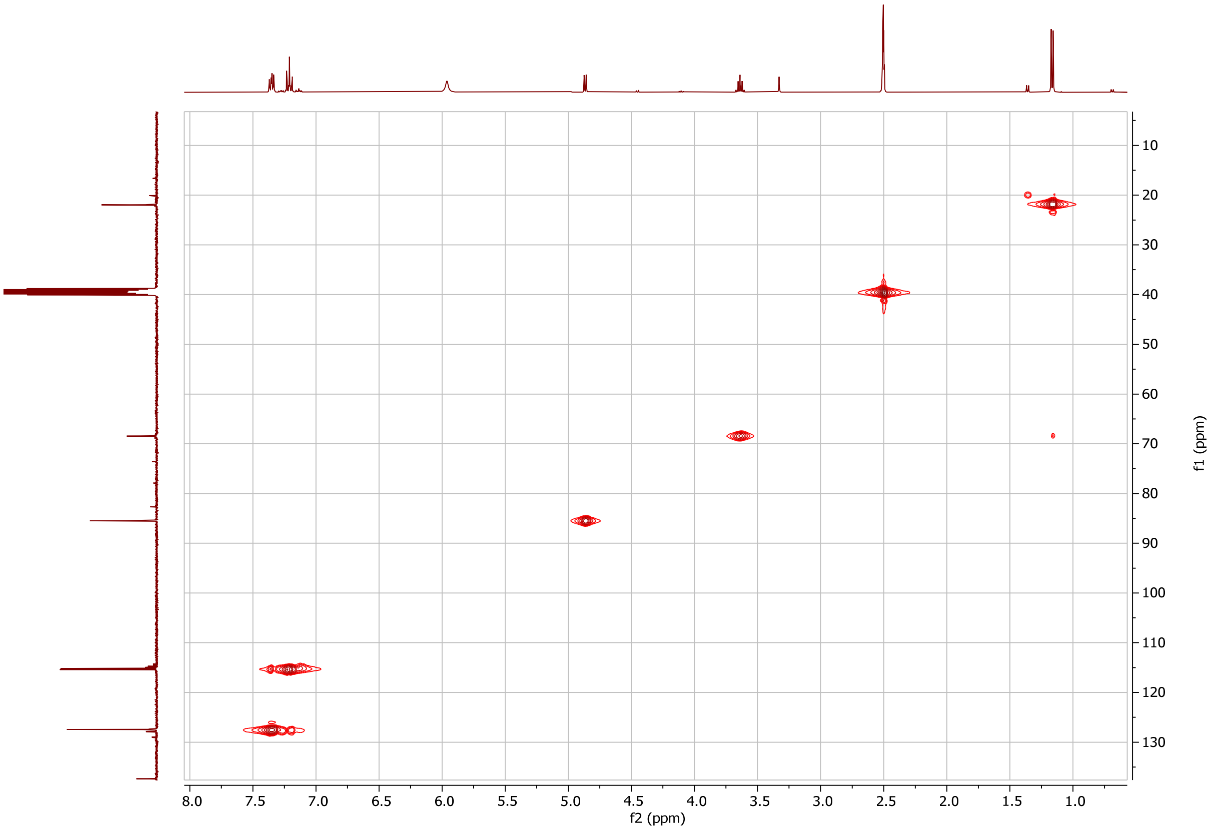

Figure S20. HSQC NMR spectrum (100/400 MHz, DMSO-d_{63}) 4F-MAR.

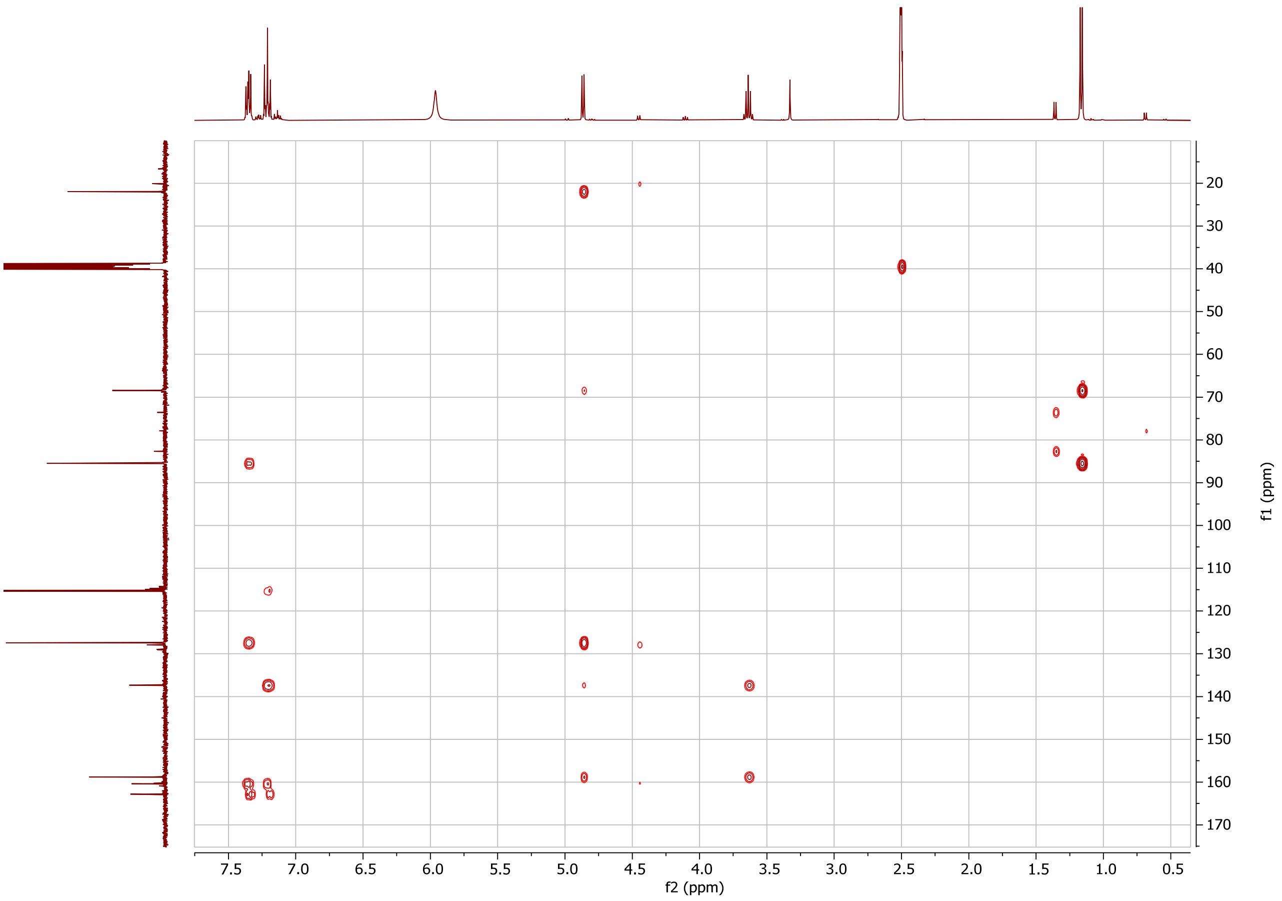

Figure S21. HMBC NMR spectrum (100/400 MHz, DMSO-d63) 4F-MAR.

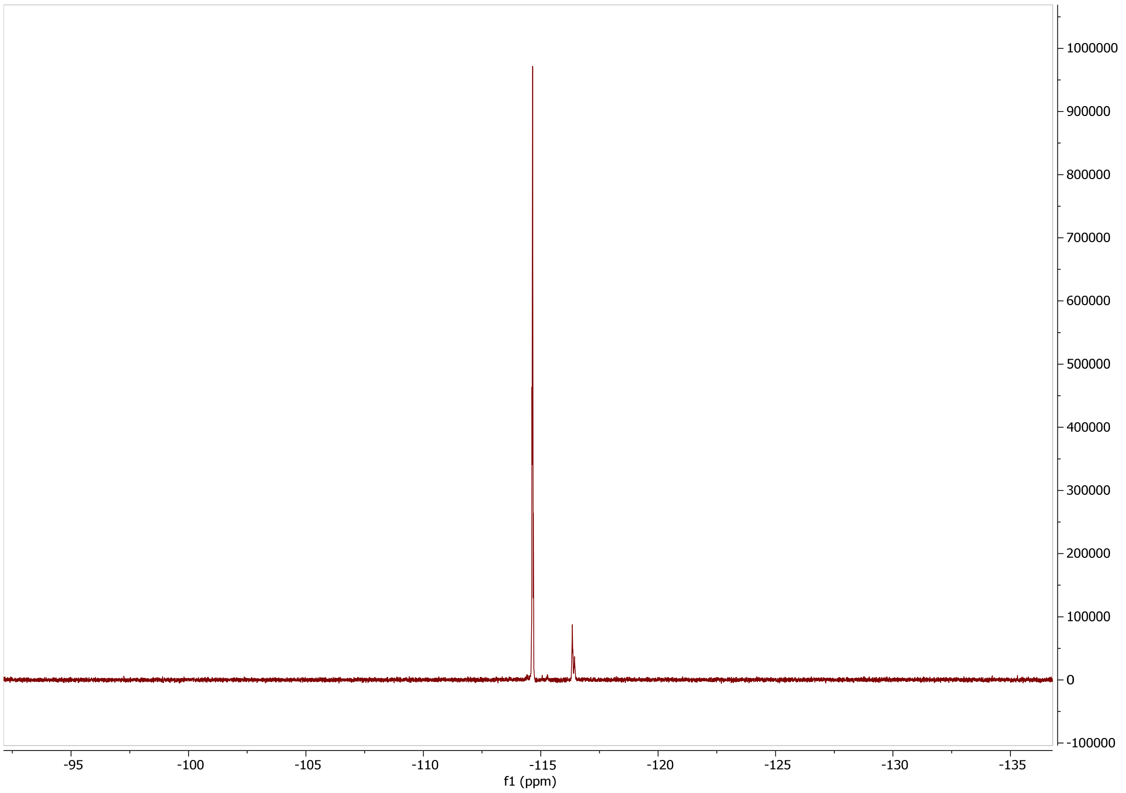

Figure S22. ^{19}F NMR spectrum (377 MHz, DMSO-d63) 4F-MAR

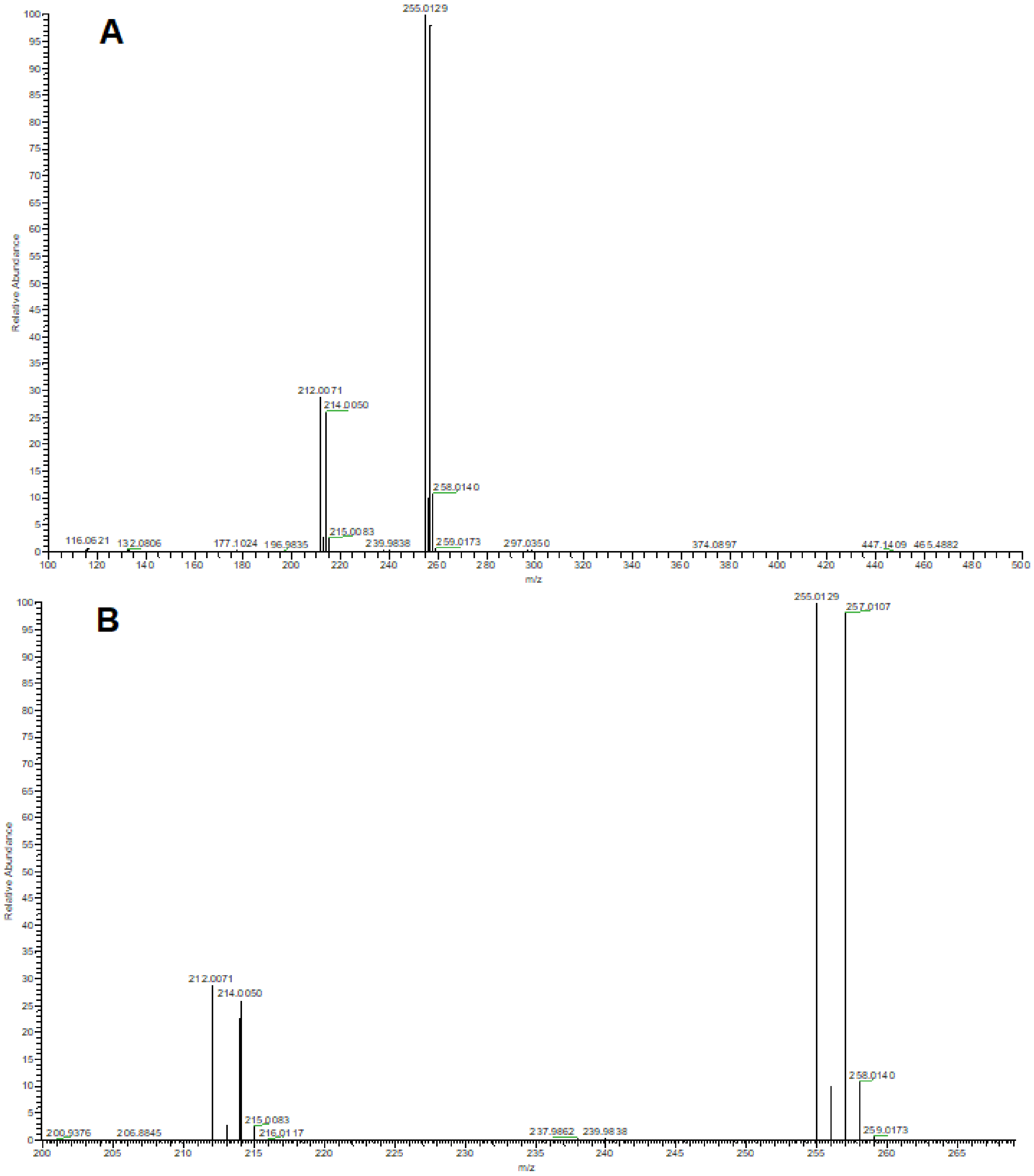

Figure S23. HESI positive mode full MS spectrum of 4B-MAR (A: full view, *m/z* 100-500, B: detailed view, *m/z* 200-270)

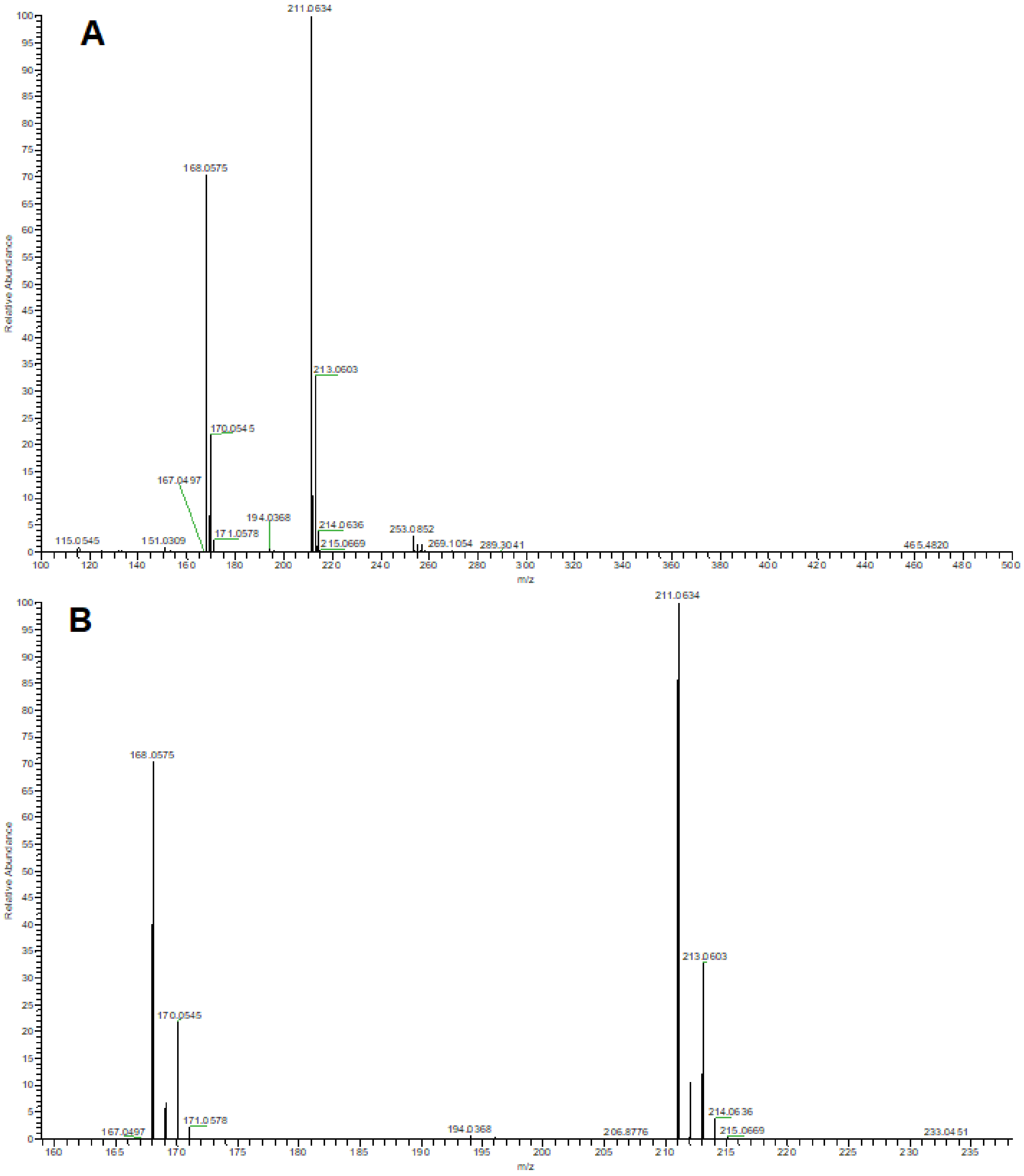

Figure S24. HESI positive mode full MS spectrum of 4C-MAR (A: full view, *m/z* 100-500, B: detailed view, *m/z* 160-240)

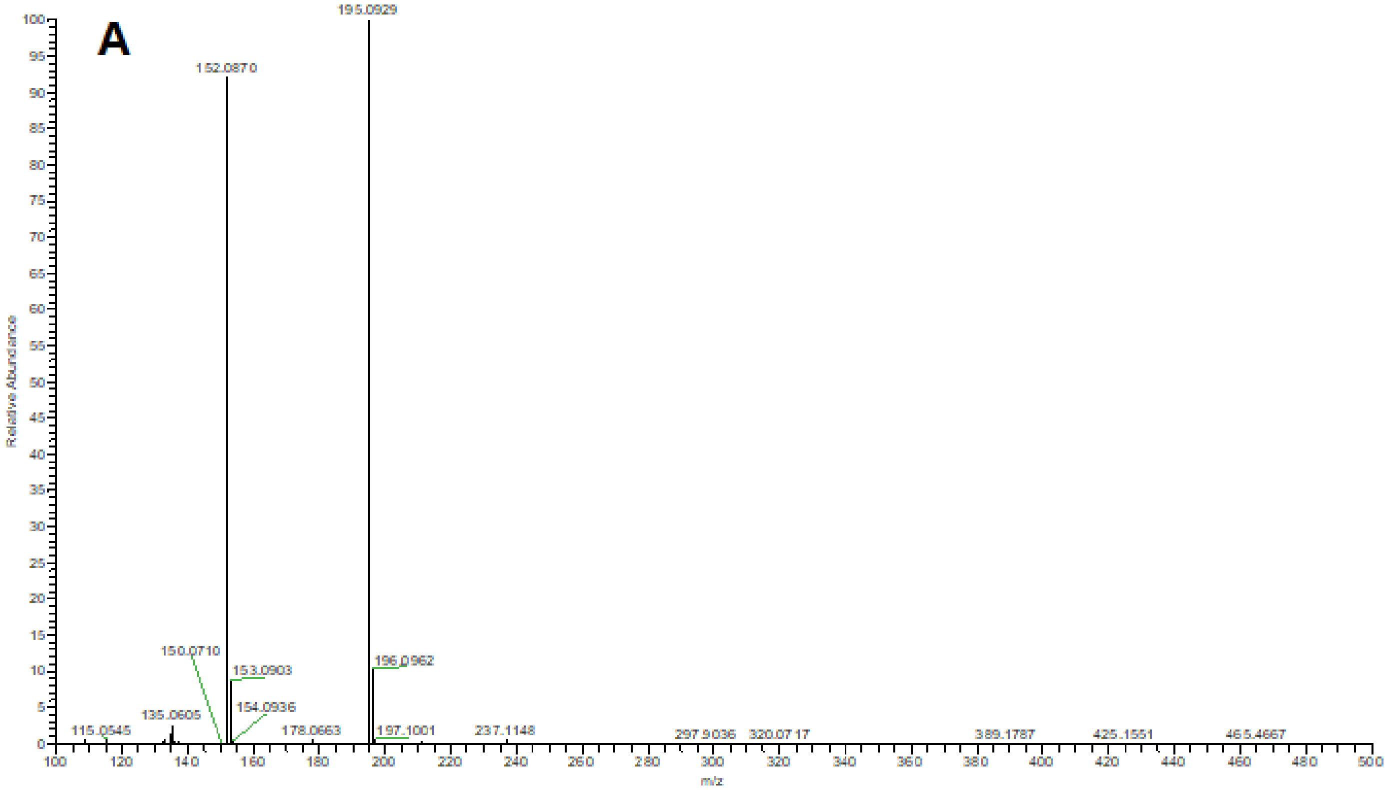

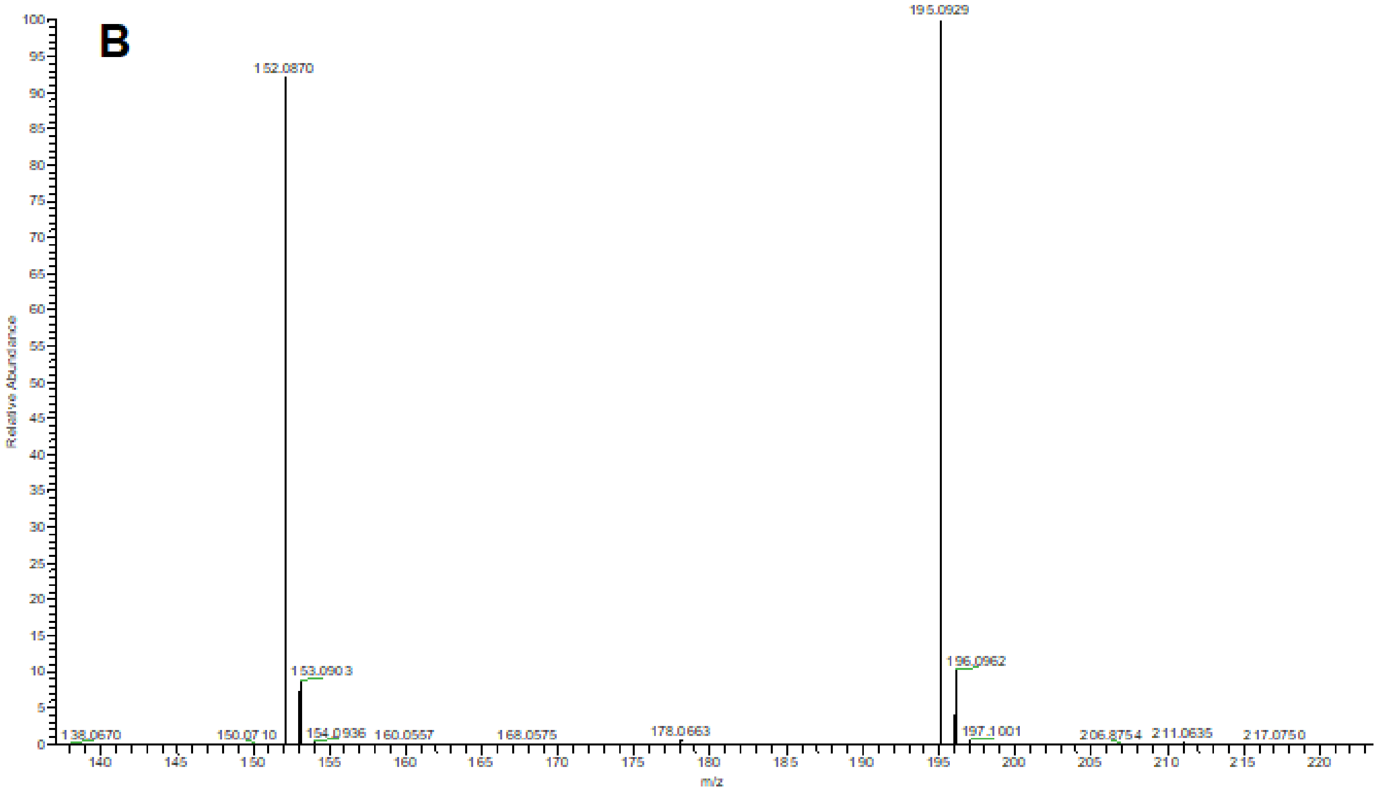

Figure S25. HESI positive mode full MS spectrum of 4F-MAR (A: full view, *m/z* 100-500, B: detailed view, *m/z* 140-220)

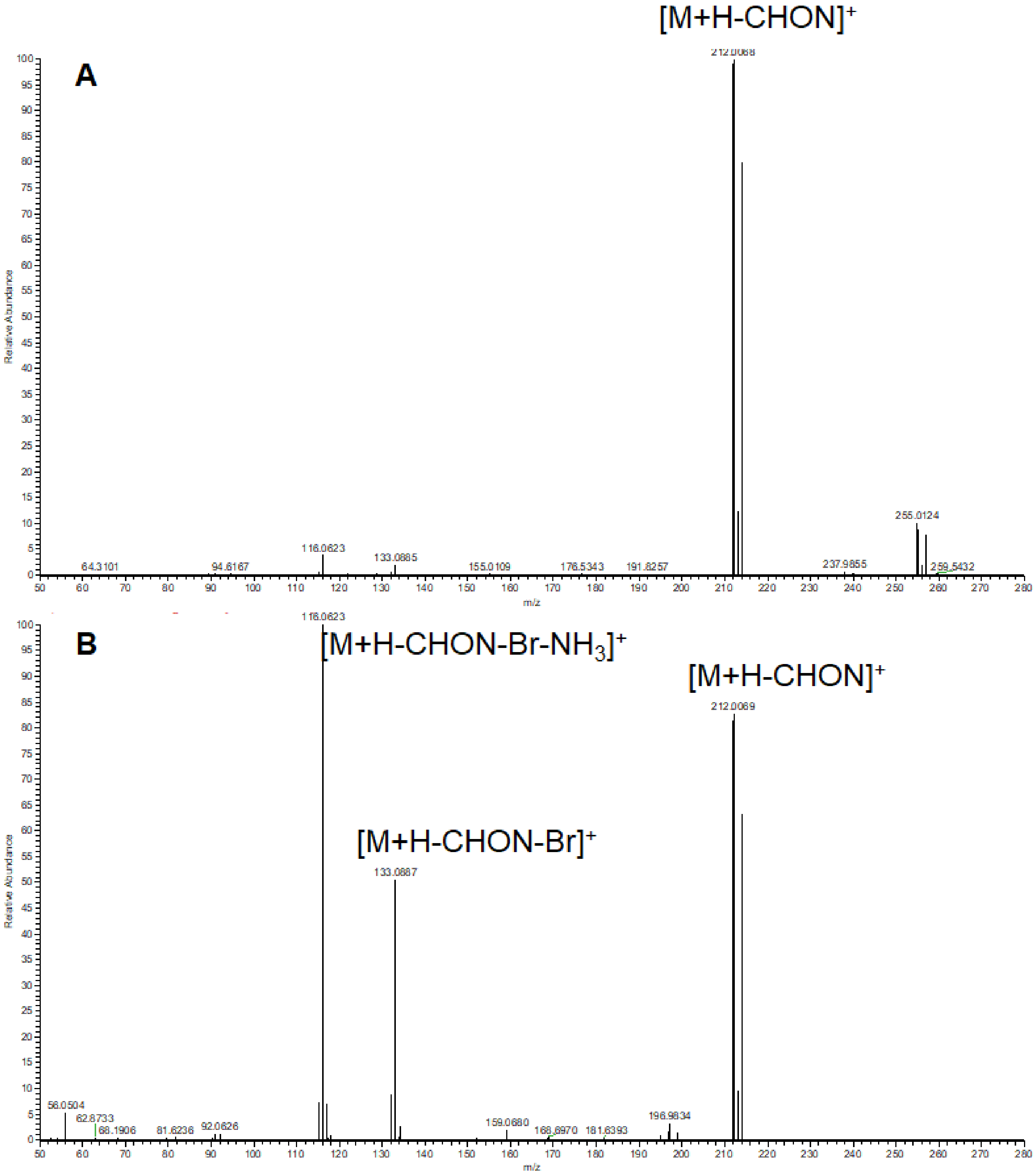

Figure S26. HESI positive mode MS/MS spectrum of 4B-MAR (A: normalized collision energy 18, B: normalized collision energy 45)

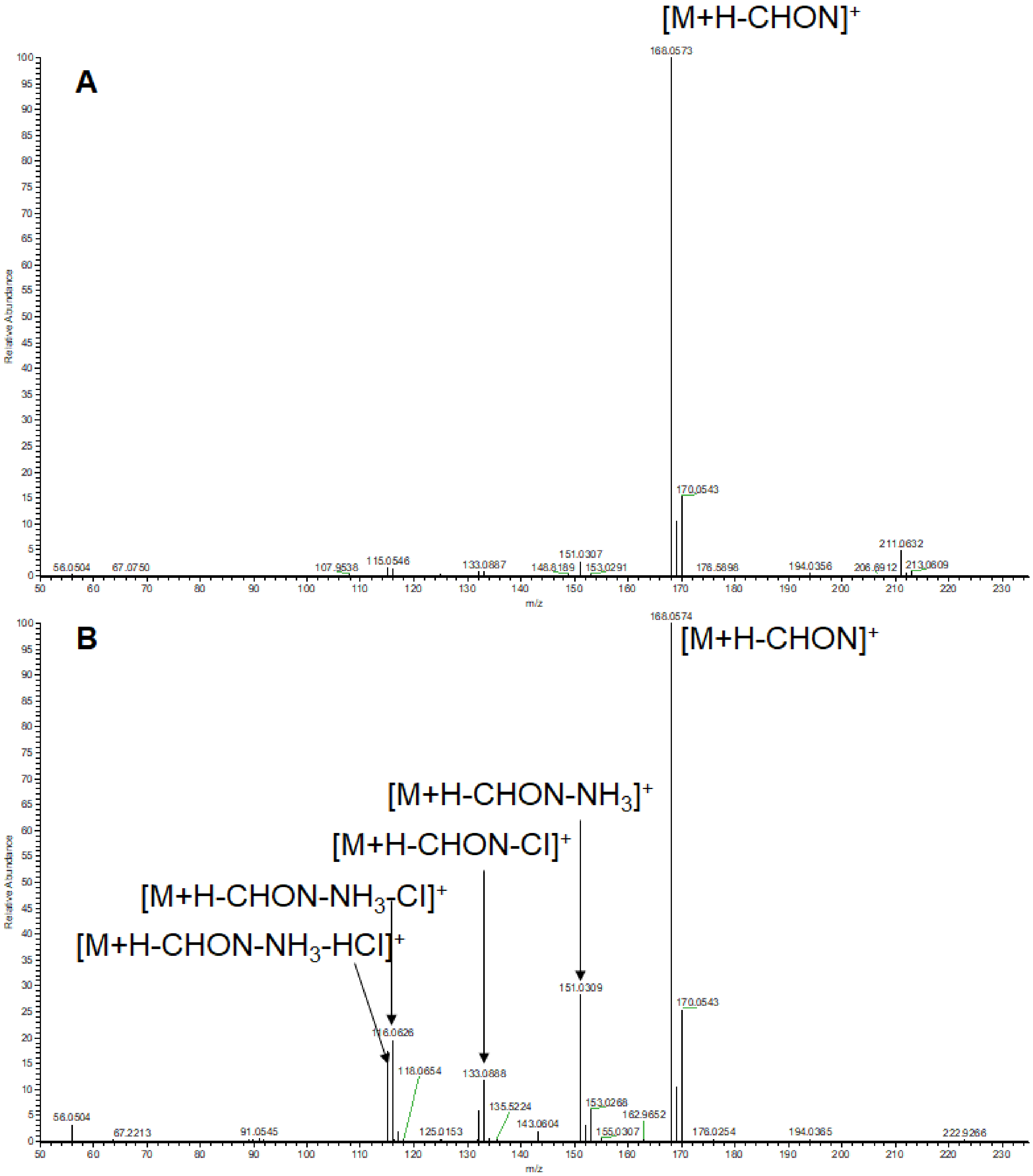

Figure S27. HESI positive mode MS/MS spectrum of 4C-MAR (A: normalized collision energy 18, B: normalized collision energy 45)

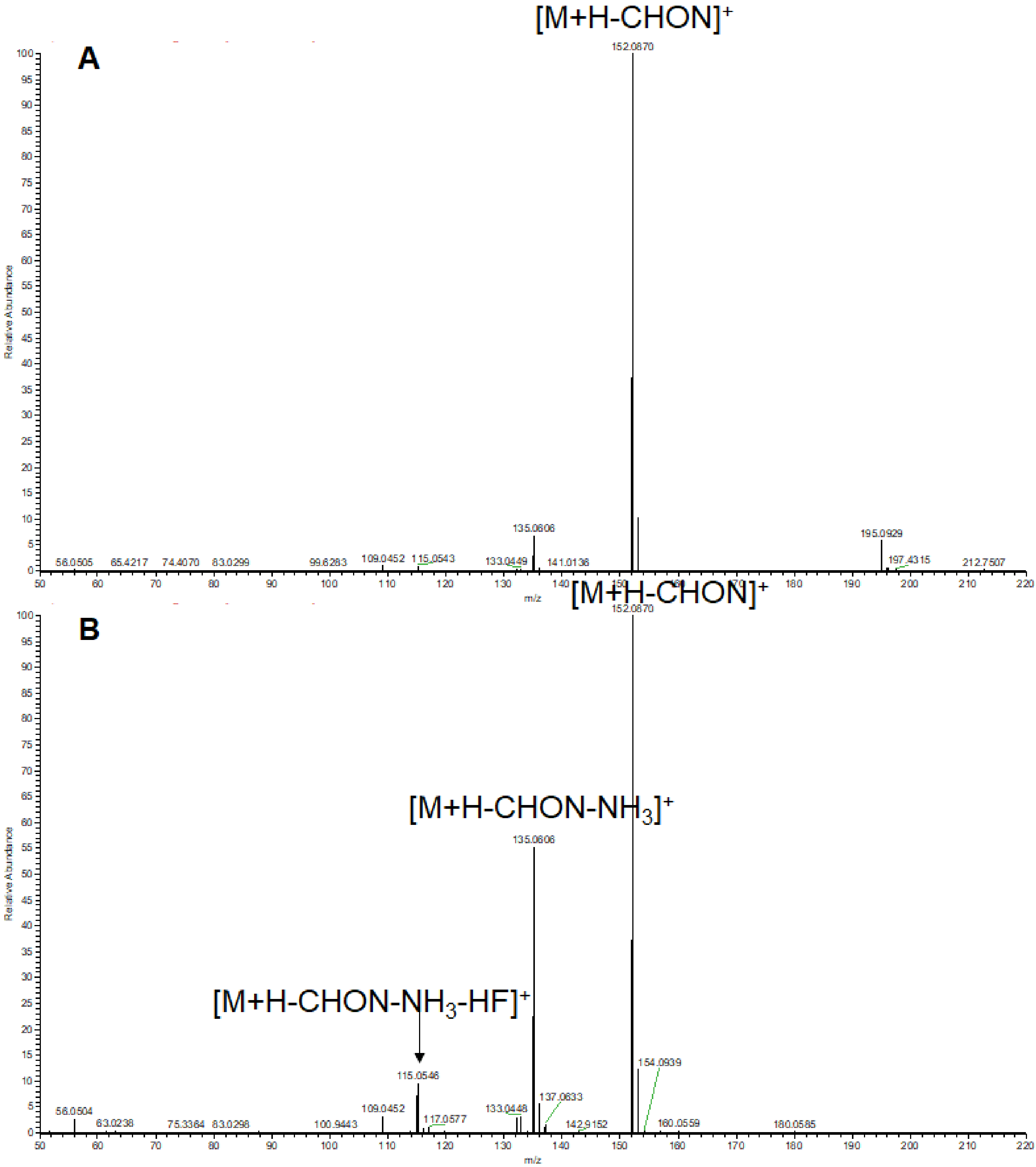

Figure S28. HESI positive mode MS/MS spectrum of 4F-MAR (A: normalized collision energy 18, B: normalized collision energy 45)
